# Taxonomic revision of New Guinea diving beetles of the *Exocelina danae* group, with the description of ten new species (Coleoptera, Dytiscidae, Copelatinae)

**DOI:** 10.3897/zookeys.619.9951

**Published:** 2016-09-27

**Authors:** Helena Shaverdo, Katayo Sagata, Michael Balke

**Affiliations:** 1 Naturhistorisches Museum, Burgring 7, A-1010 Vienna, Austria; 2 Papua New Guinea Institute for Biological research (PNG-IBR), Goroka, Papua New Guinea; 3 SNSB-Zoologische Staatssammlung München, Münchhausenstraße 21, D-81247 Munich, Germany and GeoBioCenter, Ludwig-Maximilians-University, Munich, Germany

**Keywords:** Exocelina danae-group, Copelatinae, Dytiscidae, new species, New Guinea

## Abstract

Ten new species of *Exocelina* Broun, 1886 from New Guinea are described: *Exocelina andakombensis* **sp. n.**, *Exocelina garaina* **sp. n.**, *Exocelina injiensis* **sp. n.**, *Exocelina kabwumensis* **sp. n.**, *Exocelina marawaga* **sp. n.**, *Exocelina posmani* **sp. n.**, *Exocelina tekadu* **sp. n.**, *Exocelina varirata* **sp. n.**, *Exocelina wareaga* **sp. n.**, and *Exocelina woitapensis* **sp. n**. All of them together with five already described species are united into the newly defined *Exocelina danae*-group (with *Exocelina miriae*-subgroup), a polyphyletic complex of related species with lateral setation on the median lobe. In the light of newly available material, all previously described species of the *Exocelina rivulus*-group are considered to belong to a single species, *Exocelina damantiensis* (Balke, 1998), which is now placed into the *Exocelina danae*-group, and three new synonyms are therefore proposed: *Exocelina madangensis* (Balke, 2001) **syn. n**., *Exocelina patepensis* (Balke, 1998) **syn. n**., and *Exocelina rivulus* (Balke, 1998) **syn. n**. *Exocelina tarmluensis* (Balke, 1998) **syn. n**. is a junior synonym of *Exocelina danae* (Balke, 1998). Redescription of *Exocelina atratus* (Balfour-Browne, 1939) is provided based on its type material. An identification key to all known species of the group is provided, and important diagnostic characters are illustrated. Data on the species distribution are given, showing that whilst most species are local endemics, *Exocelina damantiensis* is extremely widely distributed.

## Introduction

This paper continues our previous studies on the New Guinea species of the genus *Exocelina* Broun, 1886 (Balke 1998, 1999, Shaverdo and Balke 2014, Shaverdo et al. 2005, 2012, 2013, 2014, 2016a, b, c). So far, the New Guinea representatives of this genus are organized into five species group: the *Exocelina rivulus*-group with four species (Balke 1998), the *Exocelina aipome*(*me*)-group with four species (Balke 1998; Balke et al. 2007), the *Exocelina ullrichi*-group with three species (Balke 1998; Shaverdo and Balke 2014), the *Exocelina broschii*-group with five species (Shaverdo et al. 2005, 2016a), and by far, the largest, with 51 species, the *Exocelina ekari*-group (Balke et al. 2007, Shaverdo et al. 2012, 2014, 2016b). In the present study, we continue to build up a species group structure of the genus that can, in our opinion, provide an important tool for species identification in highly diverse genera. *Exocelina* is one of these, with 98 species described from New Guinea and 154 worldwide, including the results of this study. The *Exocelina danae*-group is defined and proposed for five already described species, together with ten new species described herein. The *Exocelina rivulus*-group was revised and abolished to avoid confusion, since all its representatives are recognized to belong to the same species, *Exocelina damantiensis* (Balke 1998), with *Exocelina rivulus* (Balke 1998) as a junior synonym. The present work also aims to provide an identification key to all treated species, as well as information about their distribution and habitats. All species data will be presented on the species-id.net portal automatically created by ZooKeys with the publication of this paper.

## Material and methods

The present work is based on the material from the following collections:

BMNH
 The Natural History Museum, London, UK 


NARI
 Papua New Guinea National Insect Collection, Port Moresby, PNG 


NHMW
 Naturhistorisches Museum Wien, Vienna, Austria 


SMNS
Staatliches Museum für Naturkunde, Stuttgart, Germany 


ZSM
 Zoologische Staatsammlung München, Munich, Germany 


All methods follow those described in details in our previous articles (Shaverdo et al. 2012, 2014, 2016b). The following abbreviations were used: TL (total body length), TL-H (total body length without head), MW (maximum body width), and hw (handwritten).

### Diagnosis of the *Exocelina danae*-group

The representatives of the *Exocelina danae*-group share the following diagnostic characters:

- beetles small or medium-sized (TL-H 3.4–4.75 mm);
- habitus oblong-oval (broadest approximately at elytral midlength), with rounded pronotal and elytral sides, body outline continuous;
- pronotum short, trapezoidal, with posterior angles not drawn backwards;
- coloration brown to piceous, mainly uniform, sometimes with paler head and pronotum and darker elytra;
- microreticulation and punctation of dorsal surface very fine to strongly impressed, beetles shiny to matt dorsally;
- metacoxae and abdominal ventrites 1–5 (and 6 in males) with thin, almost longitudinal striae/strioles;
- pronotum and elytra without striae or strioles;
- pronotum with lateral bead;
- antennomeres not modified or modified: antennomere 2 distinctly enlarged in male and female;
- male protarsomeres 1–3 not expanded laterally;
- male protarsomere 4 cylindrical, narrow, with large or small anterolateral hook-like seta;
- male protarsomere 5 not modified: long and narrow, without expansion and concavity, ventrally with two rows of short setae or with anterior band and posterior row of relatively long setae;
- median lobe of aedeagus with continuous outline in ventral and lateral views;
- ventral sclerite of median lobe more or less deeply divided apically;
- distal part of median lobe with lateral setae;
- paramere with or without notch on dorsal side;
- paramere with subdistal setae dense, strong, long; proximal setae similar to subdistal but sparser and thinner, often weakly visible.

Based on analyses of the molecular data (Toussaint et al. 2014, supplementary figs 1–4), we state that the *Exocelina danae*-group is a polyphyletic complex of the related species, most of which together with the *Exocelina broschii*-group and *Exocelina monae* (Balke, 1998) build a monophyletic cluster of morphologically diverse species with some general characters: presence of lateral pronotal bead and lateral setation of the median lobe, unmodified paramere, with distinct, dense subdistal setae and inconspicuous proximal setae, and antennomere 2 distinctly enlarged or equal to or larger than antennomere 3.

In the *Exocelina danae*-group, the *Exocelina miriae*-subgroup is recognized based on the distinctly enlarged antennomere 2. This subgroup includes three species: *Exocelina miriae* (Balke, 1998), *Exocelina rufa* (Balke, 1998), and *Exocelina tekadu* Shaverdo & Balke, sp. n. In former species, antennomere 2 is enlarged in both males and females (less strongly). The females of two latter species are unknown, therefore, we can only assume the modification of the female antennomere 2 in them. This state is also recorded for *Exocelina ullrichi* (Balke, 1998), which also has an enlarged antennomere 2 in both sexes. This is an interesting fact, since, in the majority of *Exocelina* species in New Guinea, males have modified antennomeres, but females do not have such modifications.

### Checklist and distribution of the species of the *Exocelina danae*-group

Abbreviations: IN – Indonesia, PNG – Papua New Guinea.

**Table:** 

*Exocelina miriae*-subgroup		
1.	*Exocelina miriae* (Balke, 1998)	PNG: Eastern Highlands, Morobe
2.	*Exocelina rufa* (Balke, 1998)	PNG: Morobe
3.	*Exocelina tekadu* sp. n.	PNG: Morobe
**Other species**		
4.	*Exocelina andakombensis* sp. n.	PNG: Morobe, Gulf
5.	*Exocelina atrata* (Balfour-Browne, 1939)	PNG: Oro (Northern)
6.	*Exocelina damantiensis* (Balke, 1998)	IN: West Papua: Teluk Wondama; Papua: Paniai, Intan Jaya, Puncak Jaya, Puncak, Pegunungan Bintang. PNG: Sandaun, Western, Madang, Enga, Western Highlands, Simbu, Eastern Highlands, Morobe
7.	*Exocelina danae* (Balke, 1998)	IN: Papua: Pegunungan Bintang. PNG: Sandaun
8.	*Exocelina garaina* sp. n.	PNG: Morobe
9.	*Exocelina injiensis* sp. n.	PNG: Morobe
10.	*Exocelina kabwumensis* sp. n.	PNG: Morobe
11.	*Exocelina marawaka* sp. n.	PNG: Eastern Highlands, Gulf
12.	*Exocelina posmani* sp. n.	PNG: Central
13.	*Exocelina varirata* sp. n.	PNG: National Capital District, Central
14.	*Exocelina wareaga* sp. n.	PNG: National Capital District, Central
15.	*Exocelina woitapensis* sp. n.	PNG: Central

## Species descriptions

### *Exocelina miriae*-subgroup

#### 1. Exocelina miriae

Taxon classificationAnimaliaColeopteraDytiscidae

(Balke, 1998)

Figs 2 , 3 , 25

Copelatus (Papuadytes) miriae Balke, 1998: 333; Nilsson 2001: 77 (catalogue).
Papuadytes miriae (Balke, 1998): Nilsson and Fery 2006: 56 (comb. n.).
Exocelina miriae (Balke, 1998): Nilsson 2007: 34 (comb. n.); Toussaint et al. 2014: supplementary figs 1–4, tab. 2.

##### Type locality.

Papua New Guinea: Morobe Province, Herzog Range, Wagau (Vagau), ca. 06°48'S; 146°48'E, ca. 1300 m a.s.l.

##### Type material studied.

*Paratype*: 1 male “Stn. No. 137”, “New Guinea: Morobe Dist., Herzog Mts., Vagau, C.4,000ft. 4-17.i.1965”, “M. E. Bacchus. B. M. 1965-120”, “Paratypus Copelatus miriae sp.n. Balke des. 1997” [red] (NHMW).

##### Additional material.

**Eastern Highlands**: 16 males, 15 females “Papua New Guinea: Eastern Highlands, Aiyura, 1670m, 5.iv.2006, 06.21.131S 145.54.398E, Balke & Sagata (PNG 32)” (NHMW, ZSM). 1 male “Papua New Guinea: Eastern Highlands, Aiyura, ditch in forest, 1670 m, 20.v.2006, 06.21.131S 145.54.398E, John & Balke (PNG 69)” (ZSM). 3 males, 1 female “Papua New Guinea: Eastern Highlands, Aiyura, creek, 1670 m, 20.v.2006, 06.21.131S 145.54.398E, John & Balke (PNG 70)” (ZSM). 12 males, 18 females “Papua New Guinea: Eastern Highlands, Onerunka, small creek, red soil /rock, 1700m, 21.v.2006, 06.20.936S 145.46.874E, John & Balke (PNG 71)” (NHMW, ZSM). 5 males, 5 females “Papua New Guinea: Eastern Highlands, Onerunka-Kainantu, 1799m, 14.i.2003, 06 20.561S 145 46.525E, K. Sagata (WB3)” (ZSM). 1 male, 4 females “Papua New Guinea: Eastern Highlands, Yoginofi-Kainantu, 1940m, 14.i.2003, 06 21.483S 145 45.281E, K. Sagata (WB4)” (ZSM). 1 male “390 DNA M Balke”, “PNG: EHL, Onerunka-Kainantu, ii.2003, Sagata, DNA M Balke: MB 390” (ZSM). 2 males, 1 female “Papua New Guinea: Eastern Highlands, Abave, small creek, 1500 m, 21.v.2006, 06.17.35S 145.37.681E, John & Balke (PNG 72)” (ZSM). 251 males, 127 females “Papua New Guinea: Eastern Highlands, Kainantu, Yoginofi, 1900m, 9.v.1994, 06.21.799S 145.45.463E, Balke & Sagata (PNG 55)” (NHMW, ZSM). 1 male “385 DNA M Balke”, “PNG: EHL, Kainantu, Yoginofi-Kainantu, ii.2003, Sagata, DNA M Balke: MB 385” (ZSM). 20 males, 14 females “Papua New Guinea: Eastern Highlands, Hogu, 1 km E Mt. Barola, 1900m, 9.v.1994, 06.17.556S 145.45.036E, Balke & Sagata (PNG 56)” (NHMW, ZSM). **Morobe**: 3 females “Stn. No. 139”, “New Guinea: Morobe Dist., Herzog Mts., Vagau, C.4,000ft. 4-17.i.1965”, “M. E. Bacchus. B. M. 1965-120” (BMNH). 1 male, 3 females “Stn. No. 140A”, “New Guinea: Morobe Dist., Herzog Mts., Vagau, C.4,000ft. 4-17.i.1965”, “M. E. Bacchus. B. M. 1965-120” (BMNH). 2 males, 1 female “Stn. No. 144”, “New Guinea: Morobe Dist., Herzog Mts., Vagau, C.4,000ft. 4-17.i.1965”, “M. E. Bacchus. B. M. 1965-120” (BMNH). 1 female “Stn. No. 150”, “New Guinea: Morobe Dist., Herzog Mts., Vagau, C.4,000ft. 4-17.i.1965”, “M. E. Bacchus. B. M. 1965-120” (BMNH). 42 males, 52 females “Papua New Guinea: Morobe, Wagau, Herzog Mts., 1150m, 19.xi.2006, 06.51.067S 146.48.068E, Balke & Kinibel (PNG 102)”, one male with a green label “DNA M.Balke 1380” (NHMW, ZSM). 6 males, 8 females “Papua New Guinea: Morobe, Herzog Mts., 1000m, 20.xi.2006, nr. 06.51.067S 146.48.068E, Balke & Kinibel (PNG 104)” (ZSM). 2 males, 3 females “Papua New Guinea: Morobe, Menyamya, 4-5h towds [towards] Aseki, 1500-2000m, 15.XI.2006, nr 07.14.956S 146.05.687E, Balke & Kinibel, (PNG 100)” (ZSM). 101 males, 54 females “Papua New Guinea: Gulf [sic!], Menyamya, Mt Inji 1700m, 14.xi.2006 nr 07.14.813S 146.01.330E Balke & Kinibel (PNG 96)”, one male with a green label “DNA M.Balke 1374” (NHMW, ZSM). 1 female “PAPUA NEW GUINEA Wau, Morobe Prov. Mt. Missim, 1500 m Coldwater Crk. 3 Nov 1985 Col. By MP Kowalski” (ZSM).

##### Diagnosis.

Beetle medium-sized (TL-H 3.9–4.5 mm); piceous, usually with brownish pronotal sides and head; shiny, with fine but evident punctation and microreticulation; pronotum with distinct lateral bead; male and female antennomere 2 distinctly enlarged, antennomeres 3–6 stout (Fig. 25); protarsomere 4 with large, strongly curved anterolateral hook-like setae; male protarsomere 5 ventrally with anterior band of ca. 60 and posterior row of 13 relatively long, thin setae (Fig. 2A); median lobe evenly curved, with slightly curved, elongate and broadly pointed apex in lateral view, evenly tapering, with rounded apex in ventral view, on both lateral sides with numerous fine setae situated linearly usually on anterior half of distal part of median lobe; paramere without notch, slightly concave on dorsal side and with dense, strong setae on subdistal part and fine proximal setae (Fig. 2B–D).

**Figures 1–2.**
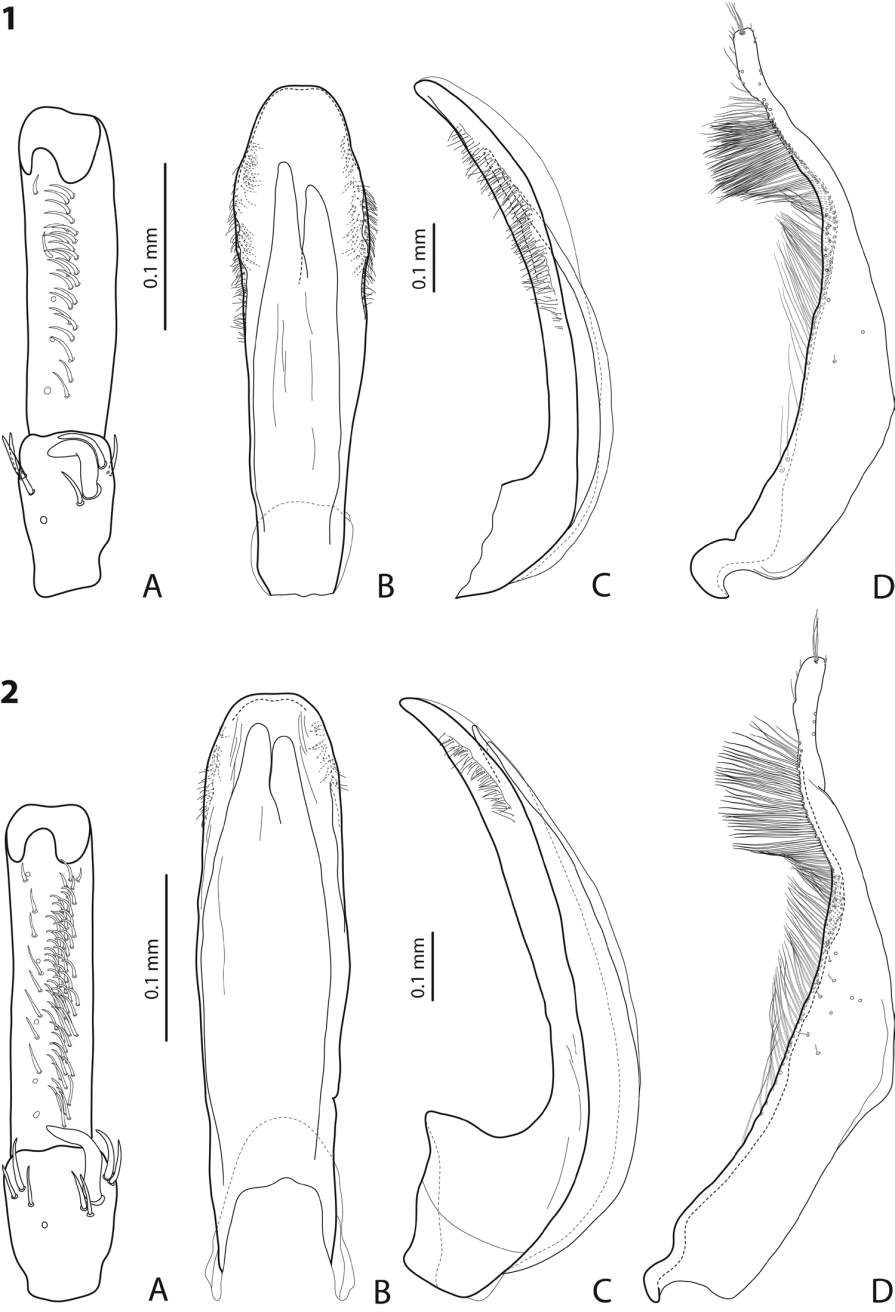
**1** *Exocelina rufa* (Balke, 1998) **2** *Exocelina miriae* (Balke, 1998), Herzog Range, Wagau **A** male protarsomeres 4–5 in ventral view **B** median lobe in ventral view **C** median lobe in lateral view **D** paramere in external view.

##### Variability.

**Figures 3–4.**
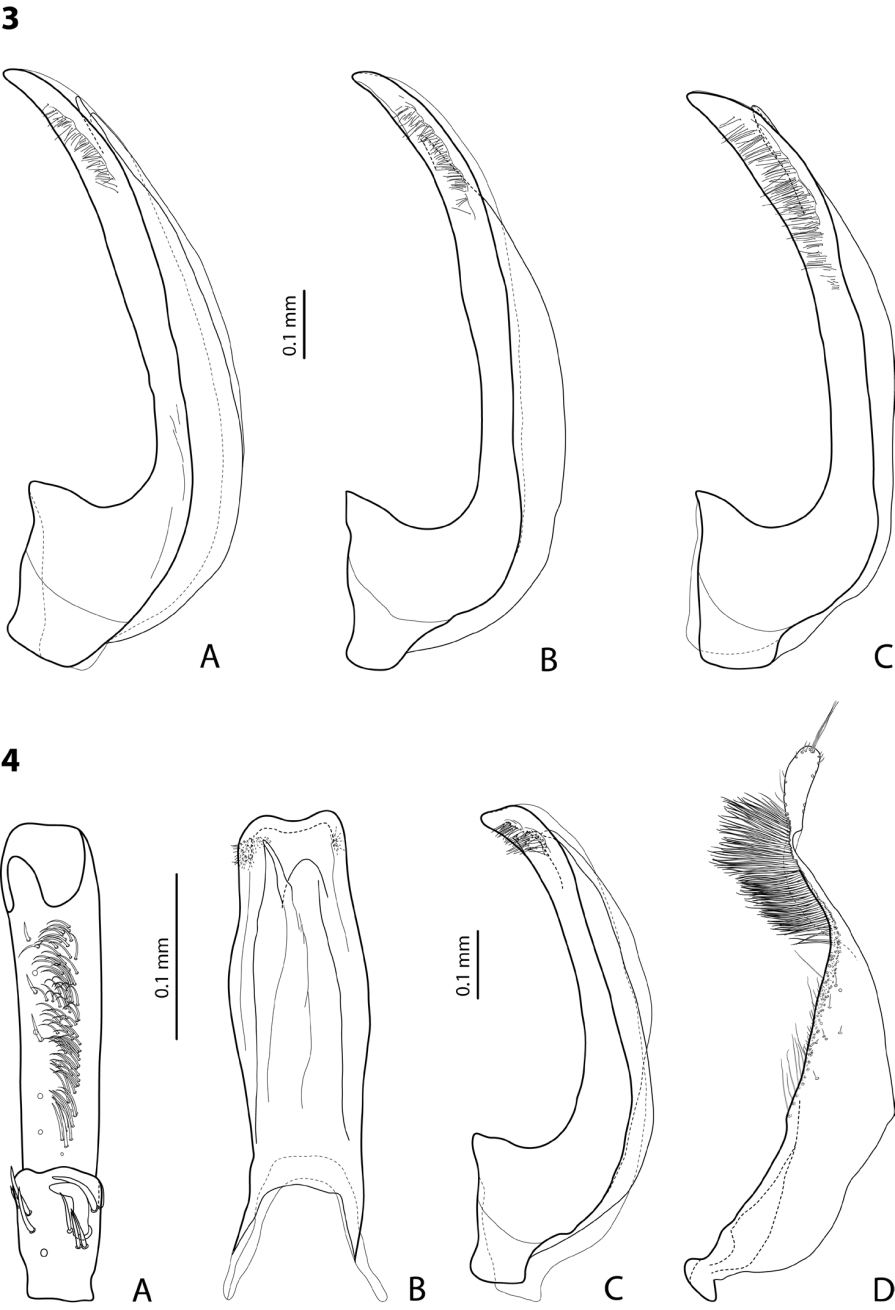
**3** *Exocelina miriae* (Balke, 1998), median lobe in lateral view **A** Herzog Range, Wagau **B** Morobe, Mount Inji, PNG96 **C** Eastern Highlands, Yoginofi, PNG55 **4** *Exocelina tekadu* sp. n. **A** male protarsomeres 4–5 in ventral view **B** median lobe in ventral view **C** median lobe in lateral view **D** paramere in external view.

Specimens from the Eastern Highlands have a shorter apex of the median lobe and more numerous lateral setae situated on almost the whole distal part of the median lobe (Fig. 3).

##### Distribution.

Papua New Guinea: Morobe and Eastern Highlands Provinces (Fig. 40).

#### 2. Exocelina rufa

Taxon classificationAnimaliaColeopteraDytiscidae

(Balke, 1998)

Figs 1 , 24

Copelatus (Papuadytes) rufus Balke, 1998: 335; Nilsson 2001: 77 (catalogue).
Papuadytes rufus (Balke, 1998): Nilsson and Fery 2006: 56 (comb. n.).
Exocelina rufa (Balke, 1998): Nilsson 2007: 34 (comb. n.).

##### Type locality.

Papua New Guinea: Morobe Province, Herzog Range, Wagau (Vagau), ca. 06°48'S; 146°48'E, ca. 1300 m a.s.l.

##### Type material studied.

*Paratypes*: 2 males “Stn. No. 150.”, “New Guinea: Morobe Dist., Herzog Mts., Vagau, C.4,000ft. 4-17.i.1965”, “M. E. Bacchus. B. M. 1965-120”, “Paratypus Copelatus rufus sp.n. Balke des. 1997” [red] (NHMW).

##### Diagnosis.

Beetle small (TL-H 3.45–3.5 mm); reddish brown; matt, with dense, strong punctation and strongly impressed microreticulation; pronotum with distinct lateral bead; male antennomere 2 distinctly enlarged, antennomeres 3–6 stout (Fig. 24); protarsomere 4 with large, strongly curved anterolateral hook-like setae; male protarsomere 5 ventrally with anterior band of 23 and posterior row of 5 relatively long, thin setae (Fig. 1A); median lobe evenly curved, with slightly curved, elongate and broadly pointed in lateral view, evenly tapering, with broadly rounded apex (slightly truncate on very tip) in ventral view, on both lateral sides with numerous fine setae situated linearly on almost whole distal part of median lobe; paramere without notch, slightly concave on dorsal side and with dense, strong setae on subdistal part and fine proximal setae (Fig. 1B–D).

##### Distribution.

Papua New Guinea: Morobe Province. The species is known only from the type locality (Fig. 40).

#### 3. Exocelina tekadu

Taxon classificationAnimaliaColeopteraDytiscidae

Shaverdo & Balke sp. n.

http://zoobank.org/E2F24500-5AB1-4AFE-B6BD-32CC3BCCC9F0

Figs 4 , 26

##### Type locality.

Papua New Guinea: Morobe Province, Tekadu, ca. 07°38'19.4"S; 146°32'12.4"E, 400–500 m a.s.l.

**Type material.** *Holotype*: male “PAPUA N.G.: Morobe Prov., Lakekamu Bas., Tekadu 28.2.1998, 400-500 m leg. Riedel” (NHMW).

##### Diagnosis.

Beetle medium-sized; brown, with reddish head and pronotum; shiny; male antennomeres modified: antennomere 2 distinctly enlarged, antennomeres 3–6 stout; protarsomere 4 with large, thick, strongly curved anterolateral hook-like seta; median lobe with slightly curved, broad apex in lateral view and with concave apex in ventral view, on both lateral sides with small bunch of fine distal setae; paramere without notch on dorsal side. The species is similar to *Exocelina miriae* and *Exocelina rufa* in the presence of the enlarged male antennomere 2, but differs from them in the shape and setation of the median lobe, as well as in distinctly finer dorsal punctation and microreticulation; from *Exocelina rufa* also in size and coloration.

##### Description.

*Size and shape*: Beetle medium-sized (TL-H 3.95 mm, TL 4.5 mm, MW 2.2 mm), with oblong-oval habitus, broadest at elytral middle. *Coloration*: Head reddish brown, with small darker areas posterior to eyes; pronotum reddish brown, with small brown to dark brown area on disc; elytra dark brown, with narrow reddish sutural lines; head appendages yellowish red, legs reddish, distally darker, especially metathoracic legs (Fig. 26).

*Surface sculpture*: Head with rather dense punctation (spaces between punctures 1–2 times size of punctures), evidently finer and sparser anteriorly; diameter of punctures smaller than diameter of cells of microreticulation or equal for some punctures. Pronotum with much sparser and finer punctation than on head. Elytra with very sparse and fine punctation. Pronotum and elytra with weakly impressed microreticulation, dorsal surface shiny. Head with microreticulation stronger. Metaventrite and metacoxa distinctly microreticulate, metacoxal plates with longitudinal strioles and transverse wrinkles. Abdominal ventrites with distinct microreticulation, strioles, and very fine sparse punctation.

*Structures*: Pronotum with distinct lateral bead. Base of prosternum and neck of prosternal process with distinct ridge, slightly rounded anteriorly. Blade of prosternal process lanceolate, relatively narrow, slightly convex, with distinct lateral bead and few setae; neck and blade of prosternal process evenly jointed. Abdominal ventrite 6 slightly truncate.

*Male*: Antennomere 2 distinctly enlarged, antennomeres 3–6 stout (Fig. 26). Protarsomere 4 with large, thick, strongly curved anterolateral hook-like seta. Protarsomere 5 ventrally with anterior band of more than 50 and posterior row of 7 rather long setae (Fig. 4A). Median lobe with slightly curved, broad apex in lateral view and with concave apex in ventral view, on both lateral sides with small number of fine setae situated in a bunch on distal part of median lobe close to apex. Paramere without notch, slightly concave on dorsal side and with dense setae on subdistal part; proximal setae inconspicuous (Fig. 4B–D). Abdominal ventrite 6 with 7–8 lateral striae on each side.

*Female*: unknown.

##### Distribution.

Papua New Guinea: Morobe Province. The species is known only from the type locality (Fig. 40).

##### Etymology.

The species is named after Tekadu Village. The name is a noun in the nominative singular standing in apposition.

## Other species

The species described below do not have modified antennae.

### 4. Exocelina andakombensis

Taxon classificationAnimaliaColeopteraDytiscidae

Shaverdo & Balke sp. n.

http://zoobank.org/91325799-1256-468B-88EB-2E19175DAF80

Figs 7 , 29

Exocelina undescribed sp. MB1361: Toussaint et al. 2014: supplementary figs 1–4, tab. 2.

#### Type locality.

Papua New Guinea: Gulf Province, Marawaka, Andakombe towards Morobe, 07°08.96'S; 145°45.48'E, 1000 m a.s.l.

#### Type material.

*Holotype*: male “Papua New Guinea: Gulf, Marawaka, Andakombe towards Morobe, 1000m, 12.xi.2006, 07.08.958S 145.45.482E, Balke & Kinibel (PNG 91)” (ZSM). *Paratypes*: **Morobe**: 1 male, 4 females “Papua New Guinea: Morobe, Herzog Mts., Bundun, 700-800m, 2.iv.2006, 06.51.598S 146.37.07E, Balke & Sagata (PNG 27)”, the male additionally with a green label “DNA M.Balke 1314” (NHMW, ZSM). **Gulf**: 2 males, 1 female with the same label as the holotype (NHMW, ZSM). 1 male “Papua New Guinea: Gulf, Marawaka, Andakombe towards Morobe, 1500m, 12.xi.2006, 07.10.413S 145.49.555E, Balke & Kinibel (PNG 93)”, “DNA M.Balke 1361” [green] (ZSM). 2 males “Papua New Guinea: Gulf, Marawaka, Mala, 1400m, 11.xi.2006, 07.05.664S 145.44.467E, Balke & Kinibel (PNG 90)” (ZSM). 3 males, 1 female “Papua New Guinea: Gulf, Marawaka, nr Ande, 1000m, 10.xi.2006, 07.03.598S 145.44.375E, Balke & Kinibel (PNG 89)” (NHMW, ZSM).

#### Diagnosis.

Beetle small; piceous, with brown head and pronotum; matt, with strong punctation and microreticulation; male antennae simple; protarsomere 4 with weakly curved anterolateral hook-like seta, equal to more laterally situated large seta; median lobe with slightly curved, rounded apex in lateral view and with slightly concave apex in ventral view, on both lateral sides with strong, relatively long setae situated broad-linearly on anterior half of distal part of median lobe; paramere without notch on dorsal side. The species is very similar to *Exocelina injiensis* sp. n. but differs from it in small, equal to laterally situated large seta, weakly curved anterolateral hook-like seta of protarsomere 4 (large, thick, strongly curved anterolateral hook-like seta in *Exocelina injiensis* sp. n.), shorter and less numerous ventral setae of protarsomere 5, and absence of fine lateral carina, bordering shorter distal setae, on the median lobe.

#### Description.

*Size and shape*: Beetle small (TL-H 3.15–3.55 mm, TL 3.55–4.1 mm, MW 1.7–1.95 mm), with oblong-oval habitus, broadest at elytral middle. *Coloration*: Head reddish brown to dark brown, with small darker areas posterior to eyes; pronotum reddish brown to dark brown, paler laterally, sometimes piceous on disc; elytra piceous, dark brown laterally, with narrow reddish sutural lines; head appendages and legs proximally yellowish red, legs distally darker, reddish brown, especially metathoracic legs (Fig. 29). Teneral specimens paler.

*Surface sculpture*: Head with very dense punctation (spaces between most of punctures equal size of punctures), sparser anteriorly; diameter of most of punctures equal diameter of cells of microreticulation. Pronotum and elytra with sparser and slightly finer punctation than on head. Pronotum and elytra with strongly impressed microreticulation, dorsal surface matt. Head with microreticulation stronger. Metaventrite and metacoxa distinctly microreticulate, metacoxal plates with longitudinal strioles and transverse wrinkles, abdominal ventrites with distinct microreticulation and strioles. Metaventrite medially, metacoxal plates, and abdominal ventrites with sparse but distinct punctation.

*Structures*: Pronotum with distinct lateral bead. Base of prosternum and neck of prosternal process with distinct ridge, slightly rounded anteriorly. Blade of prosternal process lanceolate, relatively broad, slightly convex, and smooth, with distinct lateral bead and few lateral setae; neck and blade of prosternal process evenly jointed. Abdominal ventrite 6 slightly truncate.

*Male*: Antennae simple. Protarsomere 4 with small (equal to laterally situated large seta), weakly curved anterolateral hook-like seta; small setae around it reduced. Protarsomere 5 ventrally with anterior row of 8 and posterior row of 3 short setae (Fig. 7A). Median lobe with slightly curved, rounded apex in lateral view and with almost truncate apex in ventral view, on both lateral sides with strong, short setae situated almost linearly on a half of distal part of median lobe. Paramere without notch, slightly concave on dorsal side and with dense setae on subdistal part; proximal setae inconspicuous (Fig. 7B–D). Abdominal ventrite 6 with 6–9 lateral striae on each side.

**Figures 5–6.**
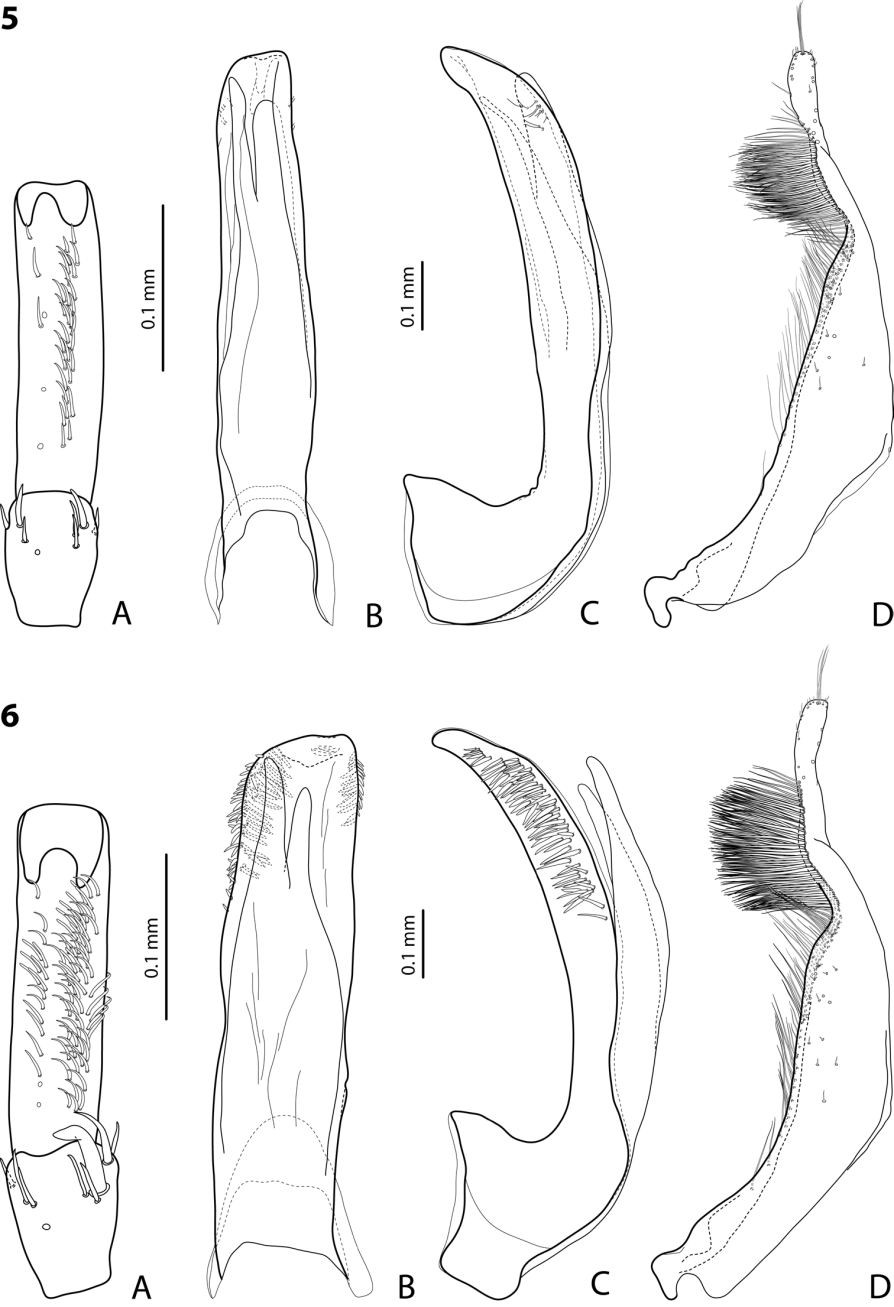
**5** *Exocelina kabwumensis* sp. n. **6** *Exocelina woitapensis* sp. n. **A** male protarsomeres 4–5 in ventral view **B** median lobe in ventral view **C** median lobe in lateral view **D** paramere in external view.

**Figures 7–8.**
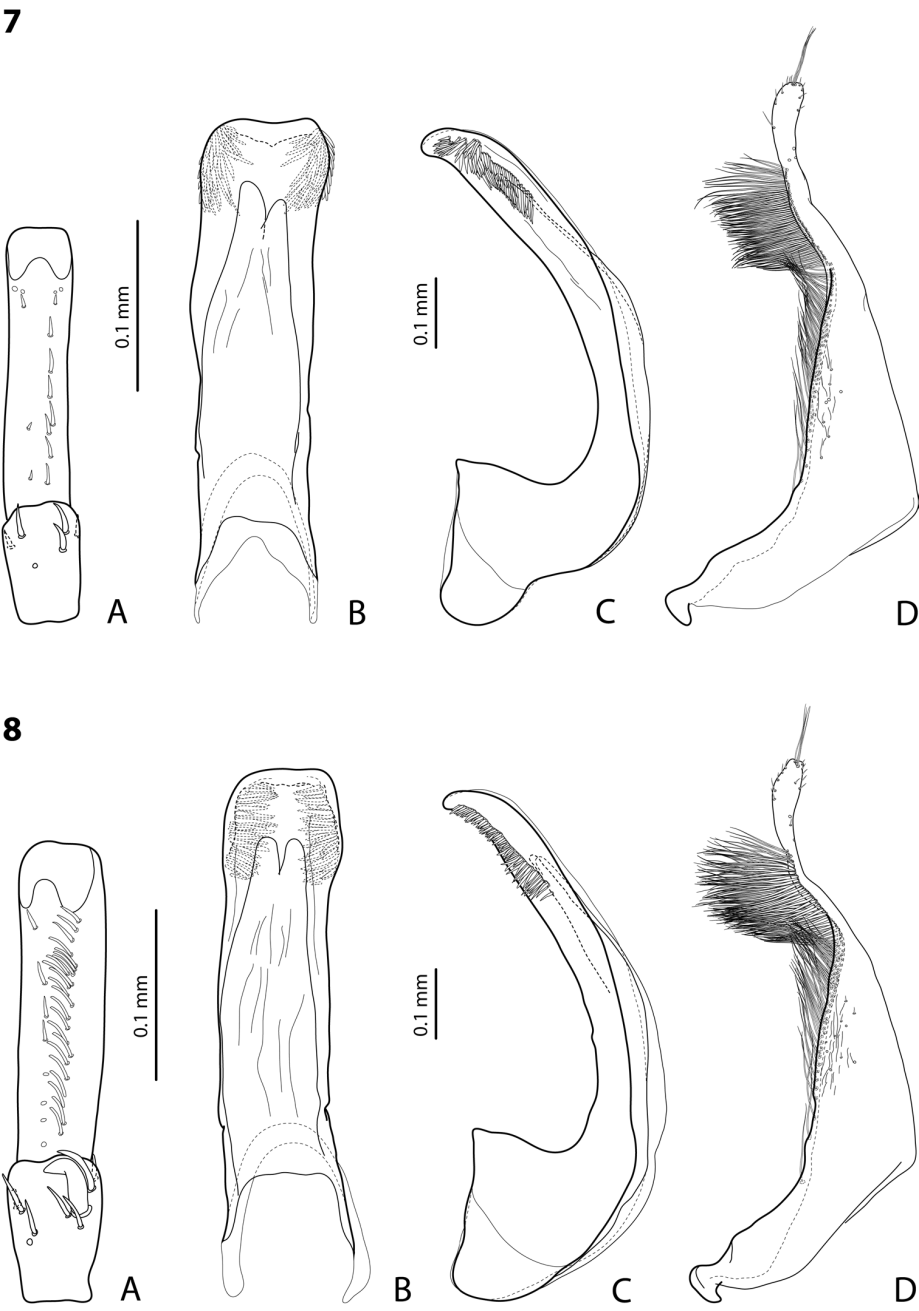
**7** *Exocelina andakombensis* sp. n. **8** *Exocelina injiensis* sp. n. **A** male protarsomeres 4–5 in ventral view **B** median lobe in ventral view **C** median lobe in lateral view **D** paramere in external view.

*Holotype*: TL-H 3.25 mm, TL 3.6 mm, MW 1.75 mm.

*Female*: Without evident differences in external morphology from males, except for not modified pro- and mesotarsi and abdominal ventrite 6 without striae.

#### Distribution.

Papua New Guinea: Gulf and Morobe Provinces (Fig. 40).

#### Etymology.

The species is named after Andakombe Village. The name is an adjective in the nominative singular.

### 5. Exocelina atrata

Taxon classificationAnimaliaColeopteraDytiscidae

(Balfour-Browne, 1939)

Figs 22 , 36

Copelatus atratus J. Balfour-Browne 1939: 66; Guignot 1956: 55 (catalogue); Guéorguiev 1968: 32 (catalogue); Guéorguiev and Rocchi 1993: 161 (catalogue).
Copelatus (Papuadytes) atratus J. Balfour-Browne, 1939: Balke 1998: 326 (notes, diagnosis); Nilsson 2001: 76 (catalogue).
Papuadytes atratus (J. Balfour-Browne, 1939): Nilsson and Fery 2006: 56 (comb. n.).
Exocelina atrata (J. Balfour-Browne, 1939): Nilsson 2007: 33 (comb. n.).

#### Type locality.

Papua New Guinea: Oro (Northern) Province, Kokoda, approximately 08°53'S; 147°44'E, approximately 366 m a.s.l.

#### Type material.

*Holotype*: male “Type” [round, with red bead], “Under stones: river side.”, “PAPUA:Kokoda. 1,200ft. viii.1933. L.E.Cheesman. B.M.1933-577.”, “Copelatus atratus, ♂ Type nov.sp.” [hw, blue ink, the word “type” with red ink], “Holotype” [red] (BMNH). *Paratypes*: 1 female “Type” [round, with red bead], “Under stones: river side.”, “PAPUA:Kokoda. 1,200ft. viii.1933. L.E.Cheesman. B.M.1933-577.”, “Copelatus atratus, ♀ Type nov.sp.” [hw, blue ink, the word “type” with red ink] (BMNH). 4 males, 1 female “Co-type” [round, with yellow bead], “Under stones: river side.”, “PAPUA:Kokoda. 1,200ft. viii.1933. L.E.Cheesman. B.M.1933-577.”, “Copelatus atratus, ♂ [or ♀] Cotype nov.sp.” [hw, blue ink, the word “Cotype” with red ink] (BMNH). 4 males “Co-type” [round, with yellow bead], “PAPUA:Kokoda. 1,200ft. vi-vii.1933. L.E.Cheesman. B.M.1933-427.”, “Copelatus atratus, B-B ♂ Co-type.” [hw, black ink] (BMNH).

#### Diagnosis.

Beetle medium-sized, dark brown, with paler, reddish-brown, head and pronotal sides; dorsal surface with fine punctation and evident microreticulation, shiny; pronotum with distinct lateral bead; male antennomeres simple; male protarsomere 4 with large, thick, strongly curved anterolateral hook-like seta; median lobe broad, with almost parallel sides and broadly rounded apex in ventral view and with slightly curved apex, some short distal setae in lateral view; paramere without notch on dorsal side.

#### Redescription.

*Size and shape*: Beetle medium-sized (TL-H 4.20–4.25 mm, TL 4.75 mm, MW 2.25 mm), with rather oblong habitus, broadest at elytral middle. *Coloration*: Head dark brown, with reddish-brown clypeus and vertex; pronotum dark brown on disc and reddish-brown on sides; elytra uniformly dark brown; ventrally pale brown to brown, slightly darker on metacoxal plates; head appendages yellowish-brown to reddish-brown, legs darker distally (Fig. 36).

*Surface sculpture*: Head with dense and coarse punctation (spaces between punctures 1–2 times size of punctures); diameter of punctures smaller than diameter of cells of microreticulation. Pronotum with evident, dense punctation, finer, sparser than on head. Elytra with finer, sparser punctation than on pronotum, punctation fine but distinct. Pronotum and elytra with distinct microreticulation, dorsal surface shiny. Head with microreticulation slightly stronger. Metaventrite, metacoxa, and abdominal ventrites distinctly microreticulate, but with cells of microreticulation larger than on dorsal side. Metacoxal plates with longitudinal strioles and transverse wrinkles; abdominal ventrites with strioles. Ventrum with inconspicuous punctation, more evident on metacoxal plates and two last abdominal ventrites.

*Structures*: Pronotum with distinct lateral bead. Base of prosternum and neck of prosternal process with distinct ridge, smooth anteriorly, without anterolateral extensions. Blade of prosternal process lanceolate, relatively narrow, convex, with distinct bead and few setae laterally; neck and blade of prosternal process evenly jointed. Abdominal ventrite 6 truncate apically.

*Male*: Antenna simple. Protarsomere 4 with large, thick, strongly curved anterolateral hook-like seta. Protarsomere 5 ventrally with dense anterior band of ca. 70 relatively long, thin setae and posterior row of 15 similar setae (Fig. 22A). Abdominal ventrite 6 with 4–5 lateral striae on each side. Median lobe broad, with almost parallel sides and slightly concave apex in ventral view and with slightly curved apex, some short distal setae situated in small groups under very fine carinas in lateral view; ventral sclerite of medial lobe as long as median lobe or slightly longer (Fig. 22B–C). Paramere without notch, slightly concave on dorsal side, with thin, sparse, inconspicuous proximal setae and thicker, denser, and longer subdistal setae (Fig. 22D).

*Female*: Without evident differences in external morphology from males, except for not modified pro- and mesotarsi and abdominal ventrite 6 rounded apically, without striae.

#### Distribution.

Papua New Guinea: Oro (Northern) Province. The species is known only from its type locality (Fig. 40).

### 6. Exocelina damantiensis

Taxon classificationAnimaliaColeopteraDytiscidae

(Balke, 1998)

Figs 9–10 , 11 , 12 , 13–14 , 15 , 16 , 31

Copelatus (Papuadytes) damantiensis Balke 1998: 314; Nilsson 2001: 76 (catalogue).
Papuadytes damantiensis (Balke, 1998): Nilsson and Fery 2006: 56 (comb. n.).
Exocelina damantiensis (Balke, 1998): Nilsson 2007: 33 (comb. n.); Toussaint et al. 2014: Supplementary Figs 1–4, Tab. 2.
Copelatus (Papuadytes) inornatus Balke 1998: 316, not Copelatus inornatus Sharp, 1882; Nilsson 2001: 77 (catalogue); **syn. n.**
Copelatus (Papuadytes) madangensis Balke 2001: 362 (nom.n. for Copelatus (Papuadytes) inornatus Balke, 1998).
Exocelina madangensis (Balke, 2001): Nilsson 2007: 34 (comb. n.); Toussaint et al. 2014: Supplementary Figs 1–4, Tab. 2.
Copelatus (Papuadytes) patepensis Balke 1998: 317; Nilsson 2001: 77 (catalogue); **syn. n.**
Papuadytes patepensis (Balke, 1998): Nilsson and Fery 2006: 56 (comb. n.).
Exocelina patepensis (Balke, 1998): Nilsson 2007: 34 (comb. n.); Toussaint et al. 2014: supplementary figs 1–4, tab. 2.
Copelatus (Papuadytes) rivulus Balke 1998: 318; Nilsson 2001: 77 (catalogue); **syn. n.**
Papuadytes rivulus (Balke, 1998): Nilsson and Fery 2006: 56 (comb. n.).
Exocelina rivulus (Balke, 1998): Nilsson 2007: 34 (comb. n.).

#### Type locality.

Papua New Guinea: Madang Province, Finisterre Range, Damanti, 05°53'26.5"S; 145°57'50.6"E.

#### Type material studied.

***Exocelina damantiensis*.** *Holotype*: male “Stn. No. 37”, “NEW GUINEA: Madang Dist., Finisterre Mts. Damanti 3,550 ft. 2-11.x.1964.”, “M.E. Bacchus. B.M. 1965-120”, “Copelatus damantiensis sp.n. Balke des. 1997” [red], “Holotypus” [red] (BMNH). Note: “Stn. 387” in the original description is obviously a type error. *Paratypes*: 2 males, 11 females “Stn. No. 37”, “NEW GUINEA: Madang Dist., Finisterre Mts. Damanti 3,550 ft. 2-11.x.1964.”, “M.E. Bacchus. B.M. 1965-120” (BMNH, NHMW). 16 males, 16 females “Stn. No. 38”, “NEW GUINEA: Madang Dist., Finisterre Mts. Damanti 3,550 ft. 2-11.x.1964.”, “M.E. Bacchus. B.M. 1965-120” (BMNH, NHMW). 3 males, 7 females, 26 exs. “Stn. No. 39”, “NEW GUINEA: Madang Dist., Finisterre Mts. Damanti 3,550 ft. 2-11.x.1964.”, “M.E. Bacchus. B.M. 1965-120” (BMNH, NHMW). 2 males, 1 female, 14 exs. “Stn. No. 61”, “NEW GUINEA: Madang Dist., Finisterre Mts. Budemu c. 4000 ft. 15-24.x.1964.”, “M.E. Bacchus. B.M. 1965-120” (BMNH, NHMW). 1 male, 1 female, 5 exs. “Stn. No. 62”, “NEW GUINEA: Madang Dist., Finisterre Mts. Budemu c. 4000 ft. 15-24.x.1964.”, “M.E. Bacchus. B.M. 1965-120” (BMNH, NHMW). 2 males, 1 female, 13 exs. “Stn. No. 73”, “NEW GUINEA: Madang Dist., Finisterre Mts. Budemu c. 4000 ft. 15-24.x.1964.”, “M.E. Bacchus. B.M. 1965-120” (BMNH, NHMW). 3 males, 11 exs. “Stn. No. 74”, “NEW GUINEA: Madang Dist., Finisterre Mts. Budemu c. 4000 ft. 15-24.x.1964.”, “M.E. Bacchus. B.M. 1965-120” (BMNH, NHMW). 6 males, 8 females “Stn. No. 78”, “NEW GUINEA: Madang Dist., Finisterre Mts. Moro.C.5550ft. 30.x.-15.xi.1964.”, “M.E. Bacchus. B.M. 1965-120” (BMNH, NHMW). 4 males, 6 females, 18 exs. “Stn. No. 82”, “NEW GUINEA: Madang Dist., Finisterre Mts. Moro.C.5550ft. 30.x.-15.xi.1964.”, “M.E. Bacchus. B.M. 1965-120” (BMNH, NHMW). 2 males, 5 exs. “Stn. No. 83”, “NEW GUINEA: Madang Dist., Finisterre Mts. Moro.C.5550ft. 30.x.-15.xi.1964.”, “M.E. Bacchus. B.M. 1965-120” (BMNH, NHMW). 2 males, 3 exs. “Stn. No. 89”, “NEW GUINEA: Madang Dist., Finisterre Mts. Moro.C.5550ft. 30.x.-15.xi.1964.”, “M.E. Bacchus. B.M. 1965-120” (BMNH, NHMW). 5 males “Stn. No. 95”, “NEW GUINEA: Madang Dist., Nr. Sewe, c.5,300 ft. 15.xi.1964.”, “M.E. Bacchus. B.M. 1965-120” (BMNH, NHMW). All these specimens are with red paratype labels “Paratypus Copelatus damantiensis sp.n. Balke des. 1997” [red].

***Exocelina madangensis*.** *Paratypes*: 4 males, 3 females with the same label as the holotype, except for “Paratypus Copelatus inornatus sp.n. Balke des. 1997” [red] (NHMW).

***Exocelina patepensis*.** *Holotype*: male “Stn. No. 126”, “NEW GUINEA: Morobe Dist., Lae-Bulolo Rd., Patep Ck., 28.xii.1964.”, “M.E. Bacchus. B.M. 1965-120”, “Holotypus” [red], “Copelatus patepensis sp.n. Balke des. 1997” [red] (BMNH). *Paratypes*: 4 males, 1 female with the same label as the holotype, except for “Paratypus Copelatus patepensis sp.n. Balke des. 1997” [red] (NHMW).

***Exocelina rivulus*.** *Holotype*: male “IRIAN JAYA, 12.8.1992 Zentralmassiv, Borme, 140°25'E 04°24'S 900m, leg. M. Balke (8)”, “HOLOTYPUS” [red], “Copelatus rivulus Balke des. 1997” [red] (NHMW). *Paratypes*: 25 males, 15 females with the same label as the holotype (NHMW). 7 males, 5 females “IRIAN JAYA: Borme ca. 140°25'E 04°24'S 950m, 3.9.1993 leg. M. Balke (2)” (NHMW). 12 males, 5 females “IRIAN JAYA Zentralmassiv 140°25'E 04°24'S”, “16.8.1992 Borme, 1000m leg. Balke (15)” (NHMW). 3 males, 5 females “IRIAN JAYA, 4.9.1992 Diuremna - Nalca 139°49'E 04°24'S 1500m, leg. Balke (36)” (NHMW). 38 males, 31 females “IRIAN JAYA, 6.9.1992 Nalca 1700-1800m 139°49'E 04°24'S leg. Balke (38)” (NHMW). 3 males, 1 female “IRIAN JAYA, 7.9.1992 Kono, 1800m 139°47'E 04°21'S, leg. Balke (41)” (NHMW). 55 males, 47 females “IRIAN JAYA, 12.9.1992 Angguruk, 1400m 139°25'E 04°15'S, leg. Balke (48)” (NHMW). 5 males, 1 female “IRIAN JAYA: Angguruk, 8.10.1993 Angguruk, ca. 1350m”, “ca. 139°25'E 04°15'S leg. M. Balke (32)” (NHMW). 1 male “IRIAN JAYA: Borme Tarmlu, 1500m 6.9.1993”, “ca. 140°25'E 04°24'S, leg. M. Balke (4-6)” (NHMW). 53 males, 42 females “IRIAN JAYA: 11.9.1993 Bime – Calab Gebiet, Bime, 1400m”, “leg. M. Balke (12) ca. 140°12'E 04°20'S” (NHMW). 57 males, 20 females “IRIAN JAYA: 22.9.1993 Bime – Calab Gebiet, Bime, 1400m”, “ca. 140°12'E 04°20'S, leg. M. Balke (16)” (NHMW). These females are a mixture of two species: *Exocelina damantiensis* and *Exocelina aipomek* (Balke, 1998). 2 males, 3 females “IRIAN JAYA: 28.9.1993 Eme Gebiet Emdoman, 1150m”, “ca. 139°55'E 04°14'S, leg. M. Balke (23)” (NHMW). 6 males, 5 females “IRIAN JAYA: 29.9.1993 Eme Gebiet Emdoman, 800m”, “ca. 139°55'E 04°14'S, leg. M. Balke (24)” (NHMW). 2 males “IRIAN JAYA: 29.9.1993 Eme Gebiet Emdoman, 800-1000m”, “leg. M. Balke (25) ca. 139°55'E 04°14'S” (NHMW). 23 males “IRIAN JAYA: 1.10.1993 Eme Gebiet Okloma, 1500m”, “ca. 139°55'E 04°14'S, leg. M. Balke (28)” (NHMW). All these specimens are with red paratype labels “PARATYPUS Copelatus rivulus sp.n. M. Balke des. 1997” or “Paratypus Copelatus rivulus sp.n. Balke des. 1997” [red].

#### Additional material.

**Indonesia: West Papua Province: Teluk Wondama Regency**: 3 males, 1 female “IRIAN JAYA: Wandammen Bay, Wondiwoi Mts. Wasior, 300-700 m, 14.I.2001 leg. A. RIEDEL” (NHMW, SMNS, ZSM). 3 females “IRIAN JAYA: Wandammen Bay, Wondiwoi Mts. Wasior, 250-600 m, 4.I.2001 leg. A. RIEDEL” (SMNS). 2 males “Indonesia: West Papua: Wandammen Bay, Wasior, 4-5.I.2001 leg. A. Riedel 2?45.940'S 134?31.738'E” (ZSM). **Papua Province: Paniai Regency**: 2 males “IRIAN JAYA: Paniai Prov. road Nabire-Ilaga, km 140 4.9.1996, 450 m leg. M. Balke (96 # 13)” (NHMW). 1 male, 5 females “IRIAN JAYA: Paniai Prov. road Nabire-Ilaga, km 160 4.9.1996, 600 m leg. M. Balke (96 # 14)” (NHMW). 1 male “IRIAN JAYA: Paniai Prov. road Nabire-Ilaga, km 165 4.9.1996, 650 m leg. M. Balke (96 # 15)” (NHMW). All these specimens (locs. 13, 14, 15) are with red paratype labels “PARATYPUS Copelatus rivulus sp.n. M. Balke des. 1997” or “Paratypus Copelatus rivulus sp.n. Balke des. 1997” [red] but they are not considered as paratypes because they are not included into the type material of the original description. **Intan Jaya Regency**: 8 males, 13 female “IRIAN JAYA: Paniai Prov. Kemandoga, Homeyo, Sabisa 1700-1900m, 5.1.1996 leg. A. Riedel” (NHMW, ZSM). **Puncak Jaya Regency**: 1 male “Indonesia: Papua, Wano Land, red clay creek nr cave, 1100m, 3.ix.2014, nr -3.587955 137.5114945 (Pap024)”, “M.Balke 6516” [green text] (ZSM). 12 males, 7 females “Indonesia: Papua, Wano Land, river grey sediment, 980m, 3.ix.2014, -3,587955 137,5114945 (Pap025)” (NHMW, ZSM). 8 males, 1 female “Indonesia: Papua, Wano Land, river ca 15m wide, 930m, 3.ix.2014, -3,587955 137,5114945 (Pap026)” (NHMW, ZSM). 6 males, 3 females “Indonesia: Papua, Wano Land, creek @ jungle helipad, 870m, 4.ix.2014, -3,584077 137,5042947 (Pap027)”, two males with an additional labels “M.Balke 6525” and “M.Balke 6526” [green text] (NHMW, ZSM). **Puncak Regency**: 11 males, 4 females “Indonesia: Papua, Wano Land, below Puluk, 1100m, 2.ix.2014, nr -3.660272 137.5207436 (Pap021)”, one of the males with an additional label “M.Balke 6510” [green text] (NHMW, ZSM). **Pegunungan Bintang Regency**: 15 males, 15 females “IRIAN JAYA Zentralmassiv 140°25'E 04°24'S”, “Kali Takime, 1000m 15.8.1992 leg. Balke (14)” (NHMW). 6 males, 7 females “IRIAN JAYA Zentralmassiv 140°25'E 04°24'S”, “Kali Takime, 900m 18.8.1992 leg. Balke (16)” (NHMW). 19 males, 27 females “IRIAN JAYA Zentralmassiv 140°25'E 04°24'S”, “Kali Takime, 900m 18.8.1992 leg. Balke (17)” (NHMW). All these specimens (locs. 14, 16, 17) are with red paratype labels “PARATYPUS Copelatus rivulus sp.n. M. Balke des. 1997” or “Paratypus Copelatus rivulus sp.n. Balke des. 1997” [red] but they are not considered as paratypes because they are not included into the type material of the original description. 9 females “IRIAN JAYA: 11.9.1993 Bime – Calab Gebiet, Bime, 1400m”, “leg. M. Balke (12) ca. 140°12'E 04°20'S” (NHMW).

**Papua New Guinea: Sandaun**: 3 females “Papua New Guinea: Sandaun, Mianmin, Fak River, 775m, 14.xi.2003, 453 53.00S 141 36 39.40E, K. Sagata (WB17)”, one of them with an additional label “DNA M. Balke 678” [green text] (ZSM). 2 females “Papua New Guinea: Sandaun, Mianmin, Fak River, 775m, 15.xi.2003, 453 53.00S 141 36 39.40E, K. Sagata (WB22)” (ZSM). 1 male “Papua New Guinea: Sandaun, Sandaun, Fak River (WB24), 23.x.2003, K. Sagata, DNA M Balke: MB 685”, “DNA M. Balke 685” [green text] (ZSM). 2 males, 5 females “Papua New Guinea: Sandaun, Sandaun, Fak River, 775m, 15.x.2003, 4 53 53.00S #, K. Sagata (WB24)” (ZSM). 1 male “Papua New Guinea: Sandaun, Sandaun, Sek River (WB50), 21.x.2003, K. Sagata, DNA M Balke: MB 668”, “DNA M. Balke 668” [green text] (ZSM). 3 males, 3 females “Papua New Guinea: Sandaun, Sandaun, Sek River 775m, 13.x.2003, K. Sagata (WB50)” (NHMW, ZSM). 5 males, 1 female “Papua New Guinea: Sandaun, May River, 970m, 19.x.2003, 4 49.779S 141 38.174E, K. Sagata (WB43)”, one of the males with an additional label “DNA M. Balke 687” [green text] (NHMW, ZSM). 2 males, 5 females “Papua New Guinea: Sandaun, Wara Uk, - 900m, 14.xi.2003, Not taken, K. Sagata (WB16)”, “DNA M. Balke 676”, “DNA M. Balke 677” [green text] (ZSM). 1 male “Papua New Guinea: Sandaun, Sandaun, Faklows (WB87), 24.x.2003, K. Sagata, DNA M Balke: MB 656”, “DNA M. Balke 656” [green text] (ZSM). 1 male “Papua New Guinea: Sandaun, Sokamin4, 1200m, 19.x.2003, 4 50.845S 141 37.865E, K. Sagata (WB102)”, “DNA M. Balke 675” [green text] (ZSM). 2 males, 2 females “Papua New Guinea: Sandaun, MekilWX25, 1718m, 13.x.2003, 4 48.637S 141 38.994E, K. Sagata (WB109)”, one of the males with an additional label “DNA M. Balke 669” [green text] (ZSM). 2 males, 1 female “Papua New Guinea: Sandaun, Mianminold, 898m, 20.x.2003, 4 53.419S 141 37.028E, K. Sagata (WB66)”, one male additionally with “DNA M. Balke 674” [green text] (ZSM). 1 female “Papua New Guinea: Sandaun, Sandaun, Mianmin (WB75), 9.x.2003, K. Sagata, DNA M Balke: MB 667”, “DNA M. Balke 667” [green text] (ZSM). 11 males, 1 female “Papua New Guinea: Sandaun, Mianmin, 670m, 22.x.2008, 4.53.329S 141.35.263E, Ibalim (PNG 189)”, one of males with an additional green label “DNA M Balke 3718” (NHMW, ZSM). 69 males, 88 females “Papua New Guinea: Sandaun, Mianmin, 670m, 20.x.2008, 4.53.292S 141.34.118E, Ibalim (PNG 191) (NHMW, ZSM). 22 males, 28 females “Papua New Guinea: Sandaun, Mianmin (river), 990m, 23.x.2008, 4.54.570S 141.35.490E, Ibalim (PNG 192)”, one of males with an additional green label “DNA M Balke 3738” (NHMW, ZSM). 8 males, 8 females “Papua New Guinea: Sandaun, Mianmin (pool), 990m, 23.x.2008, 4.54.570S 141.35.490E, Ibalim (PNG 193) (NHMW, ZSM). 51 males, 85 females “Papua New Guinea: Sandaun, Mianmin (river), 1080m, 24.x.2008, 04.55.780S 141.38.185E, Ibalim (PNG 195), some of them with green labels “DNA M Balke” with numbers 3743, 3744, 3779, 3780, 3781, 3782 (NHMW, ZSM). 21 males, 4 females “Papua New Guinea: Sandaun, Mianmin (pool), 1080m, 24.x.2008, 04.55.780S 141.38.185E, Ibalim (PNG 196)”, one of males with an additional green label “DNA M Balke 3748” (NHMW, ZSM). 82 males, 82 females “Papua New Guinea: Sandaun, Mianmin (pool), 700m, 21.x.2008, 04.52.858S 141.31.706E, Ibalim (PNG 197) (NHMW, ZSM). 27 males, 43 females “Papua New Guinea: Sandaun, Mianmin (pool), 700m, 21.x.2008, 04.52.858S 141.31.706E, Ibalim (PNG 198) (ZSM). 7 males, 15 females “Papua New Guinea: Sandaun, Mianmin area, >1000m, 23.xii.209, Ibalim & Pius (PNG232)” (ZSM). 9 males, 15 females “Papua New Guinea: Sandaun, Mianmin area, >1000m, 23.xii.2009, Ibalim & Pius (PNG240)” (NHMW, ZSM). 2 males, 2 females “Papua New Guinea: Sandaun, Mianmin area, >1000m, 26.xii.209, Ibalim & Pius (PNG233)” (ZSM). 1 male “Papua New Guinea: Sandaun, Mianmin area, >600m, 13.i.2010, Ibalim & Pius (PNG236)”, “DNA M. Balke 4928” [green text] (ZSM). 8 males, 9 females “Papua New Guinea: Sandaun, Mianmin area, >600m, 13.i.2010, Ibalim & Pius (PNG236)” (NHMW, ZSM). 11 males, 7 females “Papua New Guinea: Sandaun, Mianmin area, >600m, 9.i.2010, Ibalim & Pius (PNG237)” (NHMW, ZSM). 7 males, 4 females “Papua New Guinea: Sandaun, Mianmin area, >600m, 6.i.2010, Ibalim & Pius (PNG239)” (NHMW, ZSM). 20 males, 12 females “Papua New Guinea: Sandaun, Mianmin area, >700m, 7.i.2010, Ibalim & Pius (PNG231)” (NHMW, ZSM). **Western Province**: 27 males, 17 females “Papua New Guinea: Western Province, Tabubil, 600m, 22.vi.2008, 05.15.673S 141.13.738E, Posman (PNG 181)” (NHMW, ZSM). **Madang**: 1 female “Stn. No. 30”, “NEW GUINEA: Madang Dist., Finisterre Mts. Damanti 3,550 ft. 2-11.x.1964.”, “M.E. Bacchus. B.M. 1965-120” (BMNH). 1 male, 9 females “Stn. No. 46”, “NEW GUINEA: Madang Dist., Finisterre Mts. Damanti 3,550 ft. 2-11.x.1964.”, “M.E. Bacchus. B.M. 1965-120” (BMNH). 2 males, 3 females, 15 exs. “Stn. No. 47”, “NEW GUINEA: Madang Dist., Finisterre Mts. Damanti 3,550 ft. 2-11.x.1964.”, “M.E. Bacchus. B.M. 1965-120”. These beetles are with paratypes labels “Paratypus Copelatus damantiensis sp.n. Balke des. 1997” but they are not considered as paratypes because they are not included into the type material of the original description (BMNH, NHMW). 2 males, 2 females “Stn. No. 49”, “NEW GUINEA: Madang Dist., Finisterre Mts. Damanti 3,550 ft. 2-11.x.1964.”, “M.E. Bacchus. B.M. 1965-120” (BMNH). 2 females “Stn. No. 61”, “NEW GUINEA: Madang Dist., Finisterre Mts. Budemu c. 4000 ft. 15-24.x.1964.”, “M.E. Bacchus. B.M. 1965-120” (BMNH). 3 females “Stn. No. 82”, “NEW GUINEA: Madang Dist., Finisterre Mts. Moro. C. 5550ft. 30.x.-15.xi.1964.”, “M.E. Bacchus. B.M. 1965-120” (BMNH). 2 males, 1 female “Stn. No. 92”, “NEW GUINEA: Madang Dist., Finisterre Mts. Moro. C. 5550ft. 30.x.-15.xi.1964.”, “M.E. Bacchus. B.M. 1965-120” (BMNH). 20 males “Papua New Guinea: Madang, Akameku - Brahmin, Bismarck Range, 750m, 25.xi.2006, 05.49.892S 145.24.491E, Balke & Kinibel (PNG 113)” (NHMW, ZSM). 34 males “Papua New Guinea: Madang, Akameku - Brahmin, Bismarck Range, 750m, 25.xi.2006, nr 05.49.307S 145.24.389E, Balke & Kinibel (PNG 114)” (NARI, NHMW, ZSM). 1 male “PAPUA NEW GUINEA Madang Pr. Below Bundi, 500 m, 26IX2002, M Balke (PNG 23), “268 DNA M Balke” [green] (ZSM). 4 males, 11 females “Papua New Guinea: Madang, below Bundi, 500 m, 26.IX.2002 Balke & Sagata (PNG023)” (NHMW, ZSM). 3 males “Papua New Guinea: Madang, Simbai area, 1200m, 10.iii.2007, 05.13.389S 144.37.285E, Kinibel (PNG 152) (ZSM). 2 males “Papua New Guinea: Madang, Simbai area, 1200m, 11.iii.2007, 05.13.333S 144.37.611E, Kinibel (PNG 153) (NHMW, ZSM). **Enga**: 8 males “Papua New Guinea: Enga, Wapanamanda, 1500m, 6.xii.2006, 05.38.105S 143.55.338E, Balke & Kinibel, (PNG 128)”, one of them with an additional green label “DNA M.Balke 1527” (NHMW, ZSM). **Western Highlands**: 7 males “Papua New Guinea: Western Highlands, Kurumul, 6 Km SW Kudjip, small stream, 1580 m, 13.vi.2006, 05.53.426S 144.36.600E, John (PNG 78)”, one of them with an additional green label “DNA M.Balke 1340” (NHMW, ZSM). 1 male “Papua New Guinea: Western Highlands, Lugup River, 1700m, 4.iii.2007, 05.17.237S 144.28.214E, Kinibel (PNG 143)” (ZSM). 7 males “Papua New Guinea: Western Highlands, Above Sendiap, 1400m, 5.iii.2007, 05.19.774S 144.28.307E, Kinibel (PNG 145)”, one of them with an additional green label “DNA M.Balke 3314” (NHNW, ZSM). 10 males “Papua New Guinea: Western Highlands, Jimi Valley, above Sendiap Station, 950m, 6.iii.2007, 05.20.587S 144.28.847E, Kinibel (PNG 147) (NHNW, ZSM). **Simbu**: 1 female “Ibisca Niugini, PNG 6-8.xi.2012 Mount Wilhelm 200m -5,739897251 145,3297424 MW0200 / P0786 Vial 09596” (ZSM). 8 females “Ibisca Niugini, PNG 3-5.xi.2012 Mount Wilhelm 700m”, “-5,731960773 145,2521667 FIT-MW700-R-5/8-d10 / Plot 18 / P1238 Vial 15969-CODYTI” (ZSM). 4 females “Ibisca Niugini, PNG 28-30.x.2012 Mount Wilhelm 700m”, “-5,731960773 145,2521667 FIT-MW700-S-7/8-d04 / Plot 15 / P1211 Vial 16189-CODYTI” (ZSM). 1 female “Ibisca Niugini, PNG 30.x.-1.xi.2012 Mount Wilhelm 700m”, “-5,731960773 145,2521667 FIT-MW700-M-3/8-d06 / Plot 13 / P1196 Vial 15980-CODYTI” (ZSM). 1 male “Ibisca Niugini, PNG 31.x.-2.xi.2012 Mount Wilhelm 700m”, “-5,731960773 145,2521667 FIT-MW700-D-4/8-d07 / Plot 4 / P1125 Vial 16045-CODYTI” (ZSM). 1 male “Ibisca Niugini, PNG 7-9.xi.2012 Mount Wilhelm 700m”, “-5,731960773 145,2521667 FIT-MW700-S-7/8-d14 / Plot 19 / P1248 Vial 15781-CODYTI” (ZSM). 2 males, 1 female “Ibisca Niugini, PNG 3-5.xi.2012 Mount Wilhelm 700m”, “-5,731960773 145,2521667 FIT-MW700-K-5/8-d10 / Plot 11 / P1182 Vial 16083-CODYTI” (NHMW, ZSM). 1 female “Ibisca Niugini, PNG 4-6.xi.2012 Mount Wilhelm 700m”, “-5,731960773 145,2521667 FIT-MW700-A-6/8-d11 / Plot 1 / P1103 Vial 07195-CODYTI” (ZSM). 1 female “Ibisca Niugini, PNG 3-5.xi.2012 Mount Wilhelm 700m”, “-5,731960773 145,2521667 FIT-MW700-Q-5/8-d10 / Plot 17 / P1230 Vial 16097-CODYTI” (ZSM). 1 female “Ibisca Niugini, PNG 29-31.x.2012 Mount Wilhelm 700m”, “-5,731960773 145,2521667 FIT-MW700-J-3/8-d05 / Plot 10 / P1172 Vial 07200-CODYTI” (ZSM). 1 male “Ibisca Niugini, PNG 29-31.x.2012 Mount Wilhelm 700m”, “-5,731960773 145,2521667 FIT-MW700-D-3/8-d05 / Plot 4 / P1124 Vial 07290-CODYTI” (ZSM). 1 female “Ibisca Niugini, PNG 29-30.x.2012 Mount Wilhelm 700m”, “-5,731960773 145,2521667 FIT-MW700-E-3/8-d05 / Plot 5 / P1132 Vial 07294-CODYTI” (ZSM). 1 male, 8 females “Ibisca Niugini, PNG 27-29.x.2012 Mount Wilhelm 700m”, “-5,731960773 145,2521667 FIT-MW700-F-2/8-d03 / Plot 6 / P1139 Vial 15944-CODYTI” (ZSM). 1 female “Ibisca Niugini, PNG 31.x.-2.xi.2012 Mount Wilhelm 700m -5,73213905 145,2568207”, “FIT-MW700-C-4/8-d07 / Plot 3 / P1117 Vial 15664-CODYTI” (ZSM). 1 male, 10 females “Ibisca Niugini, PNG 27-29.x.2012 Mount Wilhelm 700m -5,731960773 145,2521667”, “FIT-MW700-D-2/8-d03 / Plot 4 / P1123 Vial 15972-CODYTI” (ZSM). 2 females “Ibisca Niugini, PNG 27-29.x.2012 Mount Wilhelm 700m -5,731960773 145,2521667”, “FIT-MW700-H-2/8-d03 / Plot 8 / P1155 Vial 15976-CODYTI” (ZSM). 3 females “Ibisca Niugini, PNG 28-30.x.2012 Mount Wilhelm 700m -5,731960773 145,2521667”, “FIT-MW700-M-2/8-d04 / Plot 13 / P1195 Vial 16167-CODYTI” (ZSM). 3 females “Ibisca Niugini, PNG 26-28.x.2012 Mount Wilhelm 700m -5,731960773 145,2521667”, “FIT-MW700-T-1/8-d02 / Plot 20 / P1250 Vial 16254-CODYTI” (ZSM). 1 male, 2 females “Ibisca Niugini, PNG 27-29.x.2012 Mount Wilhelm 700m -5,731960773 145,2521667”, “FIT-MW700-E-2/8-d03 / Plot 5 / P1131 Vial 15937-CODYTI” (ZSM). 1 female “Ibisca Niugini, PNG 25-27.x.2012 Mount Wilhelm 700m -5,731960773 145,2521667”, “FIT-MW700-D-1/8-d01 / Plot 4 / P1122 Vial 15947-CODYTI” (ZSM). 1 male, 1 female “Ibisca Niugini, PNG 3-5.xi.2012 Mount Wilhelm 700m -5,731960773 145,2521667 MW700 / P1222 Vial 16098” (ZSM). 1 female “Ibisca Niugini, PNG 3-5.xi.2012 Mount Wilhelm 700m -5,731960773 145,2521667 MW700 / P1254 Vial 16105” (ZSM). 1 male “Ibisca Niugini, PNG 5-7.xi.2012 Mount Wilhelm 700m -5,731960773 145,2521667 MW700 / P1247 Vial 16078” (ZSM). 1 male, 4 females “Ibisca Niugini, PNG 28-30.x.2012 Mount Wilhelm 700m -5,731960773 145,2521667 MW700 / P1243 Vial 16156” (ZSM). 1 male, 7 females “Ibisca Niugini, PNG 28-30.x.2012 Mount Wilhelm 700m -5,731960773 145,2521667 MW700 / P1235 Vial 16164” (ZSM). 6 females “Ibisca Niugini, PNG 26-28.x.2012 Mount Wilhelm 700m -5,731960773 145,2521667 MW700 / P1210 Vial 16172” (ZSM). 1 female “Ibisca Niugini, PNG 6-8.xi.2012 Mount Wilhelm 700m -5,731960773 145,2521667 MW700 / P1144 Vial 15649” (ZSM). 1 female “Ibisca Niugini, PNG 30.x.-1.xi.2012 Mount Wilhelm 700m -5,731960773 145,2521667 MW700 / P1220 Vial 15992” (ZSM). 1 female “Ibisca Niugini, PNG 26-28.x.2012 Mount Wilhelm 700m -5,731960773 145,2521667 MW700 / P1178 Vial 16181” (ZSM). 1 male “Ibisca Niugini, PNG 30.x.-1.xi.2012 Mount Wilhelm 700m -5,731960773 145,2521667 MW700 / P1244 Vial 16285” (ZSM). 5 females “Ibisca Niugini, PNG 28-30.x.2012 Mount Wilhelm 700m -5,731960773 145,2521667 MW700 / P1179 Vial 16186” (ZSM). 2 females “Ibisca Niugini, PNG 26-28.x.2012 Mount Wilhelm 700m -5,731960773 145,2521667 MW700 / P1226 Vial 16196” (ZSM). 1 male “Ibisca Niugini, PNG 1-3.xi.2012 Mount Wilhelm 700m -5,731960773 145,2521667 MW700 / P1237 Vial 16231” (ZSM). 2 females “Ibisca Niugini, PNG 1-3.xi.2012 Mount Wilhelm 700m -5,731960773 145,2521667 MW700 / P1213 Vial 16236” (ZSM). 6 females “Ibisca Niugini, PNG 26-28.x.2012 Mount Wilhelm 700m -5,731960773 145,2521667 MW700 / P1234 Vial 16270” (ZSM). 3 females “Ibisca Niugini, PNG 28-30.x.2012 Mount Wilhelm 700m -5,731960773 145,2521667 MW700 / P1227 Vial 16277” (ZSM). 1 male, 1 female “Ibisca Niugini, PNG 26-28.x.2012 Mount Wilhelm 700m”, “-5,731960773 145,2521667 FIT-MW700-S-1/8-d02 / Plot 19 / P1242 Vial 16118-CODYTI” (ZSM). 1 female “Ibisca Niugini, PNG 3-5.xi.2012 Mount Wilhelm 700m”, “-5,731960773 145,2521667 FIT-MW700-S-5/8-d10 / Plot 19 / P1246 Vial 16092-CODYTI” (ZSM). 1 female “Ibisca Niugini, PNG 27-29.x.2012 Mount Wilhelm 700m”, “-5,731960773 145,2521667 FIT-MW700-I-2/8-d03 / Plot 9 / P1163 Vial 15933-CODYTI” (ZSM). 1 female “Ibisca Niugini, PNG 7-9.xi.2012 Mount Wilhelm 700m”, “-5,731960773 145,2521667 FIT-MW700-P-7/8-d14 / Plot 16 / P1224 Vial 15796-CODYTI” (ZSM). 5 females “Ibisca Niugini, PNG 27-29.x.2012 Mount Wilhelm 700m”, “-5,731960773 145,2521667 FIT-MW700-A-2/8-d03 / Plot 1 / P1099 Vial 15960-CODYTI” (ZSM). 1 female “Ibisca Niugini, PNG 9-11.xi.2012 Mount Wilhelm 700m”, “-5,731960773 145,2521667 FIT-MW700-P-8/8-d16 / Plot 16 / P1225 Vial 16066-CODYTI” (ZSM). 1 female “Ibisca Niugini, PNG 31.x.-2.xi.2012 Mount Wilhelm 1200m”, “-5,720873833 145,2694702”, “FIT-MW1200-E-4/8-d07 / Plot 5 / P1523 Vial 17348” (ZSM). **Simbu/Eastern Highlands**: 3 males “Papua New Guinea: Crater Mountain, trek Haia - Wara Sera, 500m, 12IX2002, Balke & Sagata, (PNG 006)” (ZSM). 4 males “Papua New Guinea: Simbu/EHPr. Crater Mountain, Wara Sera Station, 800 m, 14IX2002, Balke & Sagata, (PNG 009)” (NHMW, ZSM). 10 males “Papua New Guinea: Crater Mountain, Wara Sera Station, 800 m, 14IX2002, Balke & Sagata (PNG 010)” (NHMW, ZSM). 1 female “Papua New Guinea: Simbu/EHPr. Crater Mountain, Sera - Herowana, Jau river, 1000 m, 15IX2002, Balke & Sagata (PNG 015)” (ZSM). 2 females “Papua New Guinea: Simbu/EHP, Crater Mountain, Sera - Herowana, Sima river, 1250 m, 15IX2002, Balke & Sagata (PNG 016)” (ZSM). 1 male “PNG Simbu / EHPr. Crater Mountain, Sera - Herowana, Wara Hulene, 1000 m, 16IX2002, Balke & Sagata (PNG 17)”, “263 DNA M Balke” [green] (ZSM). 8 males, 5 female “Papua New Guinea: Simbu / EHPr. Crater Mountain, Sera - Herowana, Hulene river, 1000 m, 16IX2002, Balke & Sagata (PNG 017)” (NHMW, ZSM). **Eastern Highlands**: 1 female “Stn. No. 182”, “NEW GUINEA: E. Highland Dist., Purosa Valley, nr. Okapa. 8.ii.1965.”, “M.E. Bacchus. B.M. 1965-120” (BMNH). 1 female “Stn. No. 190”, “NEW GUINEA: E. Highland Dist., Okapa, c. 5.000ft. 10-11.ii.1965.”, “M.E. Bacchus. B.M. 1965-120” (BMNH). 11 males “Papua New Guinea: Eastern Highlands, Akameku - Brahmin, Bismarck Range, 700m, 24.xi.2006, 05.52.754S 145.23.209E, Balke & Kinibel (PNG 109)”, one of them with an additional green label “DNA M.Balke 1519” (NHMW, ZSM). 20 males “Papua New Guinea: Eastern Highlands, Akameku - Brahmin, Bismarck Range, 800m, 24.xi.2006, 05.50.021S 145.24.664E, Balke & Kinibel (PNG 112)” (NARI, NHMW, ZSM). 2 males, 1 female “Papua New Guinea: Eastern Highlands, below Yonki, 850m, 4.iv.2006, 06.11.332S 146.03.052E, Balke & Sagata (PNG 31)”, one male additionally with “DNA M.Balke 1311” [green] (ZSM). **Morobe**: 5 females “Stn. No. 112”, “NEW GUINEA: Morobe Dist., Finisterre Mts. Hinggia, c. 2,500ft. 28.xi.1964.”, “M.E. Bacchus. B.M. 1965-120” (BMNH). 16 males, 25 females “Papua New Guinea: Morobe, Huon, 1 km E Yakop, 1400m, 14.v.2006, nr 06.10.961S 147.08.204E, Sagata (PNG 74)” (NHMW, ZSM). 74 males, 24 females “Papua New Guinea: Morobe, Huon, Dalasi, 3 km N Yakop, 1900m, 15.v.2006, 06.10.961S 147.08.204E, Sagata (PNG 75)”, “DNA M.Balke 1286” [green] (NHMW, ZSM). 1 male “PNG: Huon Peninsula, Morobe Prov., Yus conservation area, 1398m.”, “DNA M.Balke 541” [green text] (ZSM). 10 males, 4 female “Papua New Guinea: Morobe, Mindik, 1480m, 10.x.2009, 06.27.335S 147.25.233E, Inaho (03) (PNG 203) (NHMW, ZSM). 6 males “Papua New Guinea: Morobe, Mindik, 1490m, 11.x.2009, 06.27.315S 147.25.166E, Inaho (04) (PNG 204) (NHMW, ZSM). 1 male “PAPUA N.G.: Morobe Prov. Mindik, 1200 – 1500 m, 26.4.1998 leg. A. Riedel” (NHMW). 63 males, 38 females “PAPUA N.G.: Morobe Prov. E Pindiu, Kobau 24.4.1998, 1400 m leg. A. Riedel” (NHMW, ZSM). 3 males, 1 female “Papua New Guinea: Morobe, Penjengjeng, 1200m, 12.x.2009, 06.27.497S 147.29.219E, Inaho (05) (PNG205)”, one male additionally with “DNA M.Balke 3822” [green] (NHMW, ZSM). 3 males, 1 female “Papua New Guinea: Morobe, Pindiu, Sulemana, 850m, 15.x.2009, 06.25.169S 147.32.112E, Inaho (08) (PNG 208)” (NHMW, ZSM). 1 male “Papua New Guinea: Morobe, Sattelberg, Maro Creek, 670m, x.2009, ca 06.27.239S 147.42.531E, Inaho (10) (PNG210)”, “DNA M.Balke 3826” [green] (ZSM). 3 males, 1 female “Papua New Guinea: Morobe, Sattelberg, Zige River, ca 700m, x.2009, 6 29.233S 147 46.482E, Inaho (12a) (PNG212)” (NHMW, ZSM). 4 males, 3 females “Papua New Guinea: Morobe, Sattelberg, Siki River, ca 700m, 20.x.2009, 6 29.352S 147 46.544E, Inaho (12c) (PNG 214)” (NHMW, ZSM). 15 males, 6 females “Papua New Guinea: Morobe, Huon Pen., Kwapsanek, 850m, 31.iii.2006, 06.34.913S 147.00.526E, Balke & Sagata (PNG 25)”, one of males with an additional green label “DNA M.Balke 1315” (ZSM).

#### Females of doubtful identity.

**Indonesia: Papua Province: Pegunungan Bintang Regency**: 3 females “IRIAN JAVA: Borme Tarmlu 1500m 6.9.1993”, “ca. 140°25'E 04°24'S leg. M. Balke (4-6)” (NHMW). 1 female “IRIAN JAVA: Borme Tarmlu 1500m 6.9.1993”, “ca. 140°25'E 04°24'S leg. M. Balke (4)” (NHMW). 2 females “IRIAN JAVA: Borme Tarmlu 1500m 6.9.1993”, “ca. 140°25'E 04°24'S leg. M. Balke (6)” (NHMW). These females are a mixture of four species: *Exocelina damantiensis*, *Exocelina ketembang* (Balke, 1998), *Exocelina aipomek* (Balke, 1998), and *Exocelina danae* (Balke, 1998). 1 male (no genitals), 27 females “IRIAN JAYA: 1.10.1993 Eme Gebiet Okloma, 1500m”, “ca. 139°55'E 04°14'S, leg. M. Balke (28)” (NHMW). These females are a mixture of three species: *Exocelina damantiensis*, *Exocelina ketembang*, and *Exocelina aipomek*. 13 females “IRIAN JAYA: 22.9.1993 Bime – Calab Gebiet, Bime, 1400m”, “ca. 140°12'E 04°20'S, leg. M. Balke (16)” (NHMW). 2 females “IRIAN JAYA, 24.-26.9.1993 Eipomek [sic!] Gebiet Eipomek [sic!] - Diruemna”, “ca. 140°01'E 04°27'S 1800-2600m, leg. M. Balke (21-22)” (NHMW). These females are a mixture of two species: *Exocelina damantiensis* and *Exocelina aipomek*. **Papua New Guinea: Madang**: 15 females “Papua New Guinea: Madang, Akameku - Brahmin, Bismarck Range, 750m, 25.xi.2006, 05.49.892S 145.24.491E, Balke & Kinibel (PNG 113)” (NHMW, ZSM). 25 females “Papua New Guinea: Madang, Akameku - Brahmin, Bismarck Range, 750m, 25.xi.2006, nr 05.49.307S 145.24.389E, Balke & Kinibel (PNG 114)” (NARI, NHMW, ZSM). These females are a mixture of two species: *Exocelina broschii* (Balke, 1998) and *Exocelina damantiensis*. 19 females “Papua New Guinea: Madang, Simbai area, 1200m, 10.iii.2007, 05.13.389S 144.37.285E, Kinibel (PNG 152)” (NHMW, ZSM). These females are a mixture of two species: *Exocelina broschii* and *Exocelina damantiensis*. 53 females “Papua New Guinea: Madang, Simbai area, 1200m, 11.iii.2007, 05.13.333S 144.37.611E, Kinibel (PNG 153)” (NARI, NHMW, ZSM). These females are a mixture of three species: *Exocelina broschii*, *Exocelina simbaiarea* Shaverdo & Balke, 2014, and *Exocelina damantiensis*. **Enga**: 10 females “Papua New Guinea: Enga, Wapanamanda, 1500m, 6.xii.2006, 05.38.105S 143.55.338E, Balke & Kinibel, (PNG 128)” (ZSM). These females are a mixture of two species: *Exocelina mondmillensis* Shaverdo, Sagata & Balke, 2016 and *Exocelina damantiensis*. **Western Highlands**: 142 females “Papua New Guinea: Western Highlands, Kurumul, 6 Km SW Kudjip, small stream, 1580 m, 13.vi.2006, 05.53.426S 144.36.600E, John (PNG 78)” (NARI, NHMW, ZSM). These females are a mixture of three species: *Exocelina mondmillensis*, *Exocelina edeltraudae* (Shaverdo, Hendrich & Balke, 2012), and *Exocelina damantiensis*. 34 females “Papua New Guinea: Western Highlands, Lugup River, 1700m, 4.iii.2007, 05.17.237S 144.28.214E, Kinibel (PNG 143)” (NHMW, ZSM). 9 females “Papua New Guinea: Western Highlands, Above Sendiap, 1400m, 5.iii.2007, 05.19.774S 144.28.307E, Kinibel (PNG 145)” (ZSM). 9 females “Papua New Guinea: Western Highlands, Jimi Valley, above Sendiap Station, 950m, 6.iii.2007, 05.20.587S 144.28.847E, Kinibel (PNG 147) (ZSM). These females are a mixture of two species: *Exocelina mondmillensis* and *Exocelina damantiensis*. **Eastern Highlands**: 12 females “Papua New Guinea: Eastern Highlands, Akameku - Brahmin, Bismarck Range, 700m, 24.xi.2006, 05.52.754S 145.23.209E, Balke & Kinibel (PNG 109)” (ZSM). 24 females “Papua New Guinea: Eastern Highlands, Akameku - Brahmin, Bismarck Range, 800m, 24.xi.2006, 05.50.021S 145.24.664E, Balke & Kinibel (PNG 112)” (NARI, NHMW, ZSM). These females are a mixture of two species: *Exocelina broschii* and *Exocelina damantiensis*.

#### Diagnosis.

**Figures 9–10.**
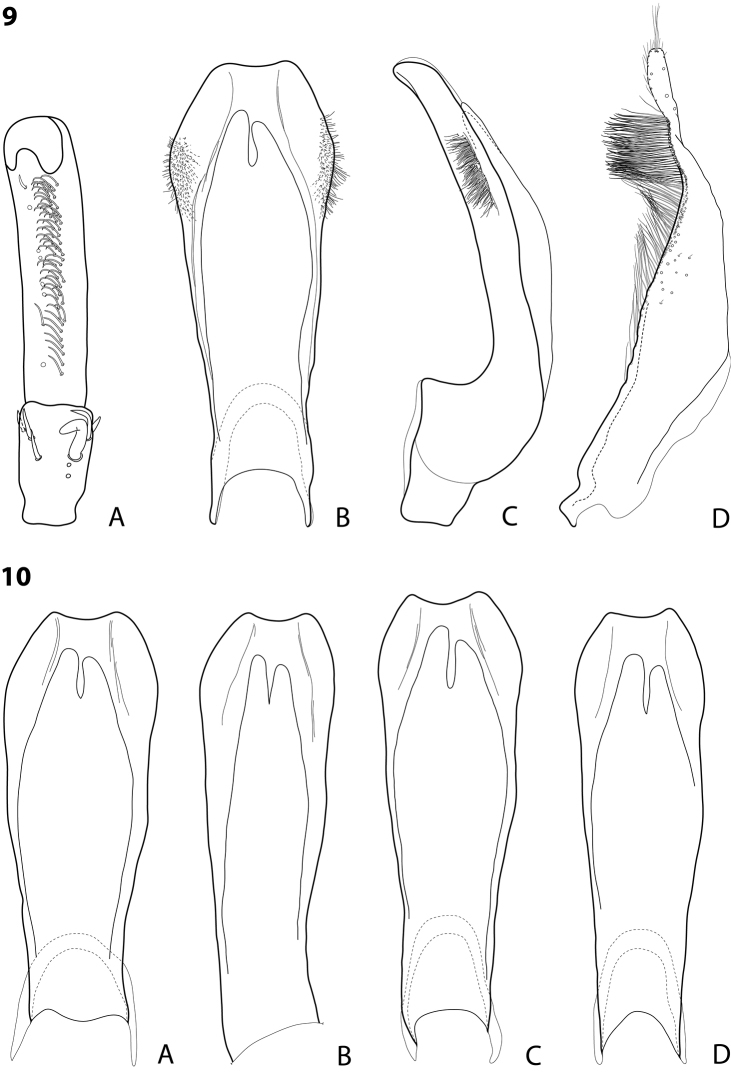
**9** *Exocelina damantiensis* (Balke, 1998), paratype, Madang, Damanti **A** male protarsomeres 4–5 in ventral view **B** median lobe in ventral view **C** median lobe in lateral view **D** paramere in external view **10** *Exocelina damantiensis*, median lobe in ventral view, setae are not shown **A** IN, West Papua, Wasior **B** IN, Papua, Nabire-Ilaga, 96#13 **C**, **D** IN, Papua, Wano, Pap027 and Pap024.

Beetle medium-sized (TL-H 3.7–4.5 mm); uniformly brown to piceous or with paler head, pronotum or only its sides, with or without reddish sutural lines on elytra; shiny, with fine punctation and microreticulation; dorsal punctation on elytra often almost invisible; pronotum with distinct lateral bead; male antennae simple (Fig. 31); protarsomere 4 with large, thick, strongly curved anterolateral hook-like seta; male protarsomere 5 ventrally with anterior band of more than 40 and posterior row of 8 relatively long, thin setae (Fig. 9A); median lobe broad, of characteristic shape in ventral view: broadened subdistally and narrowed apically, with slightly to distinctly concave apex, in lateral view, with curved, slightly elongate and broadly pointed apex, on both lateral sides with fine setae situated on distal part of median lobe under very fine carinas; paramere without notch on dorsal side and dense, long subdistal setae and inconspicuous proximal setae (Fig. 9B–D). See also the original descriptions in Balke (1998). The species can be easily mixed up with some occurring species: the shiny species of the *Exocelina broschii*-group, *Exocelina broschii* and *Exocelina mondmillensis*, or with *Exocelina ketembang* and *Exocelina aipomek*, from which can be reliably distingused only by the shape of the median lobe.

#### Variability and notes on species delimitation.

**Figure 11.**
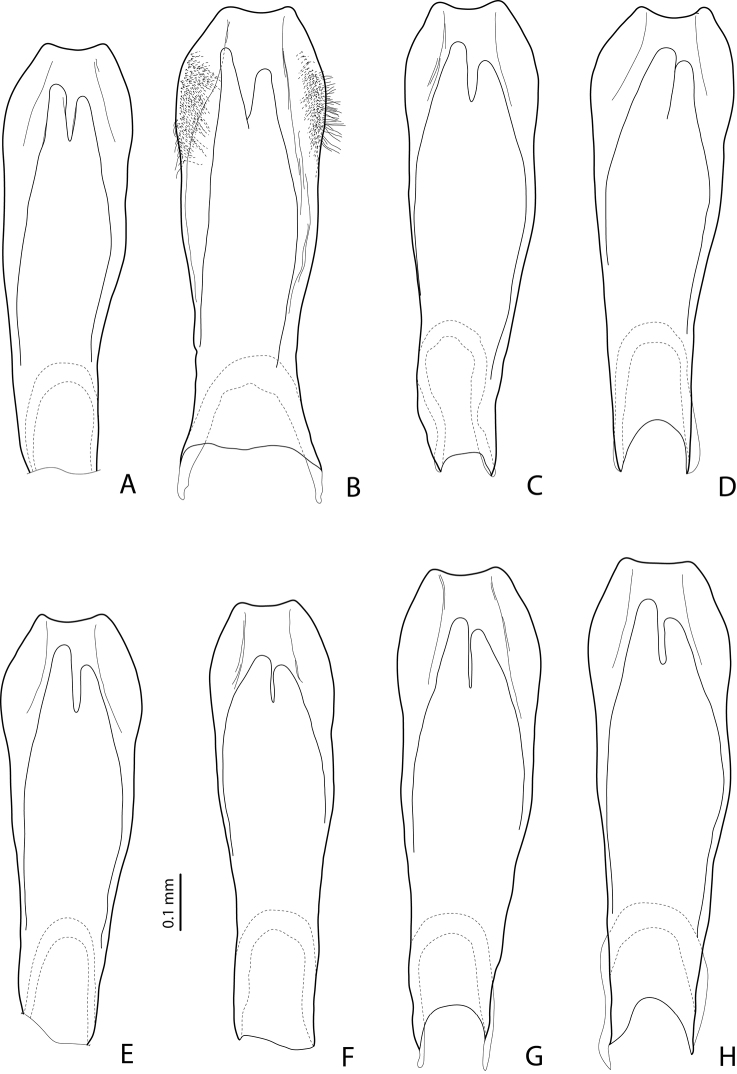
*Exocelina damantiensis* (Balke, 1998), median lobe in ventral view, setae are not shown **A** IN, Papua, Bime-Calab, 16, paratype of *Exocelina rivulus* (Balke, 1998) **B** IN, Papua, Angguruk, 32, paratype of *Exocelina rivulus* **C, D** PNG, Sandaun, Mianmin area, PNG236 and Wara-Uk, WB16 **E, F** PNG, Western Province, Tabubil, PNG181 **G** PNG, Enga, PNG128 **H** PNG, WHL, PNG147.

**Figure 12.**
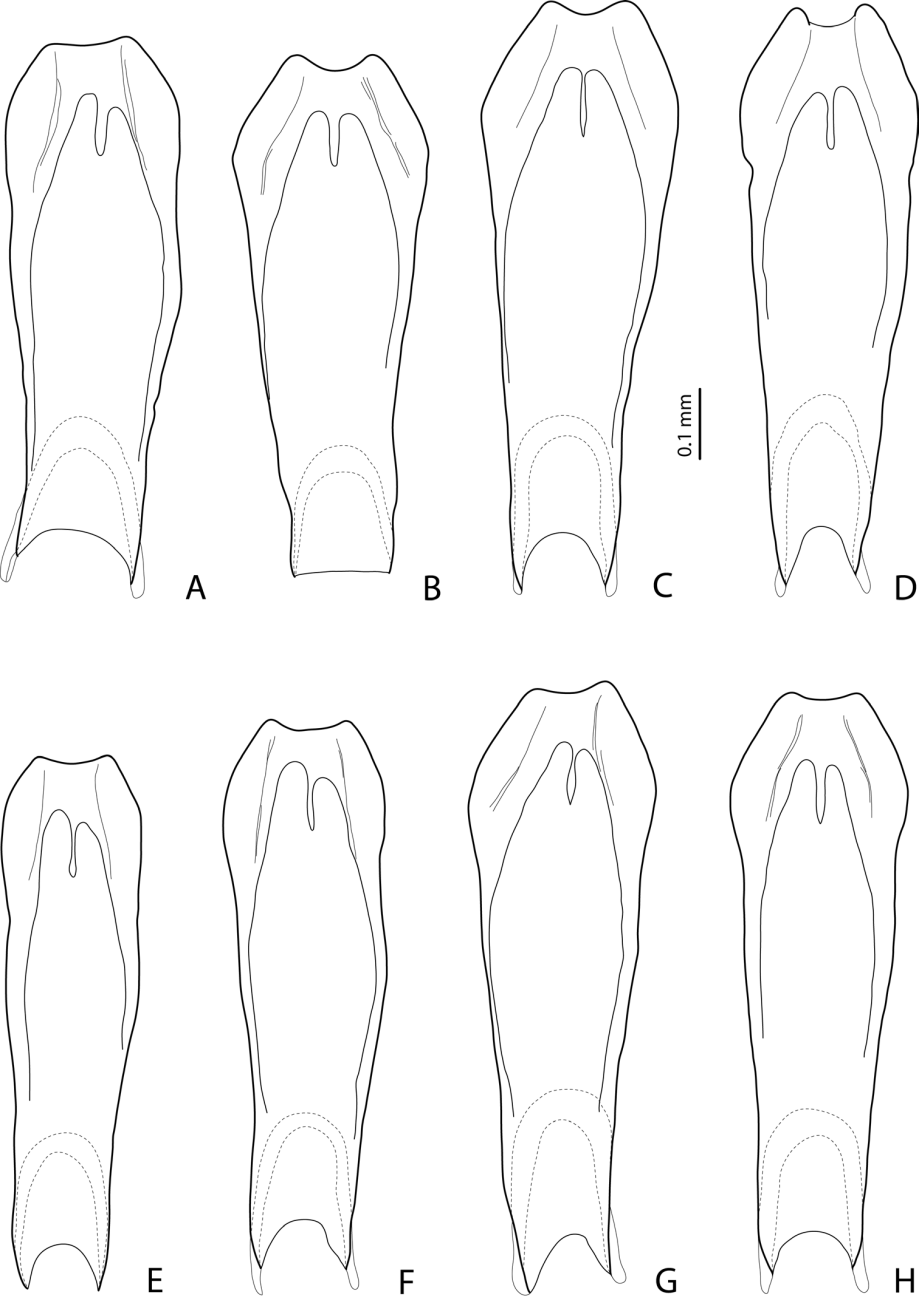
*Exocelina damantiensis* (Balke, 1998), PNG, median lobe in ventral view, setae are not shown **A** Simbu, Mount Wilhelm **B** Simbu/EHL, Wara Sera, PNG10 **C, D** Simbu/EHL, Hulene River, PNG17 **E** Madang, Brahman-Bundi, paratype of *Exocelina madangensis* (Balke, 2001) **F** Madang, Akameku-Brahman, PNG114 **G, H** Madang, Damanti, paratypes of *Exocelina damantiensis*.

**Figures 13–14.**
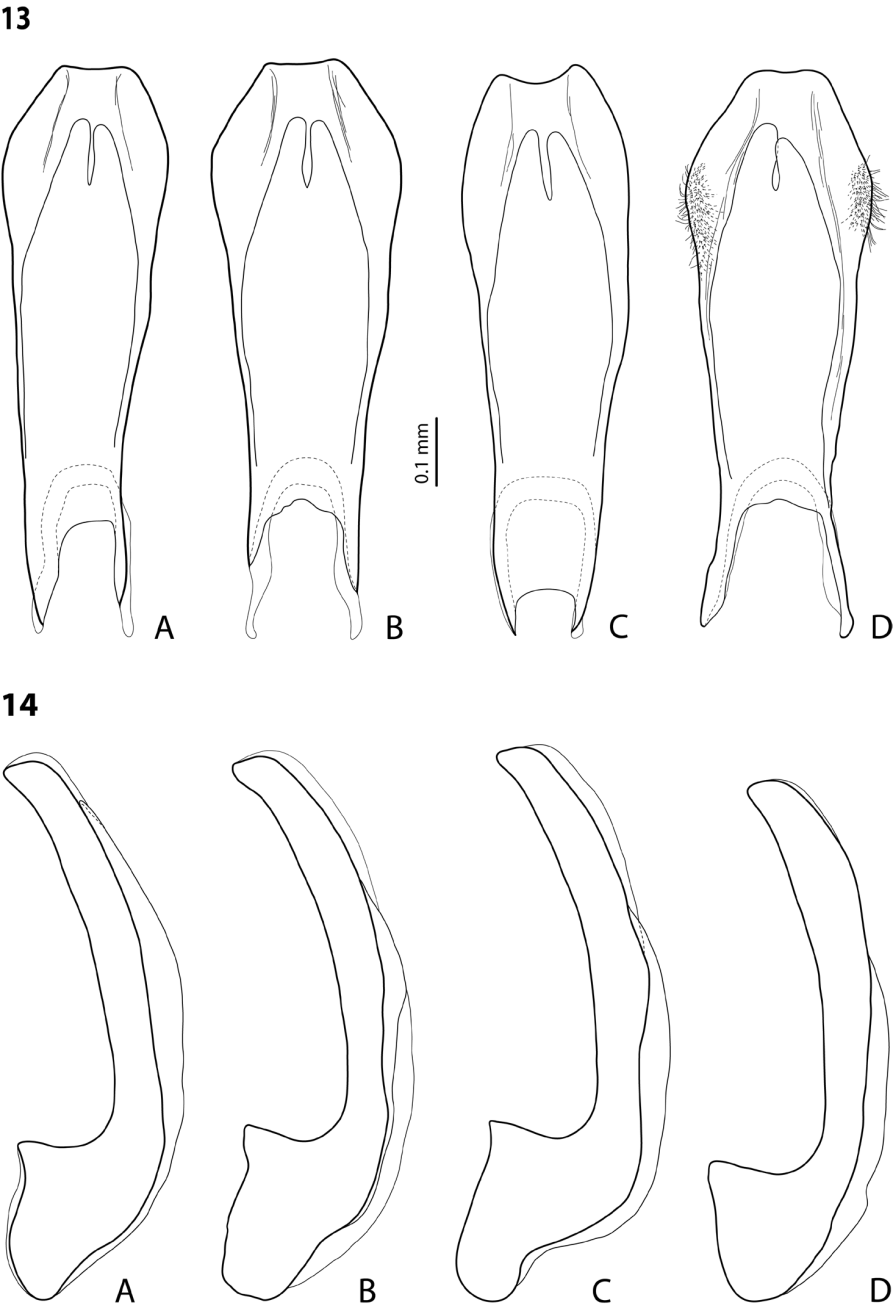
*Exocelina damantiensis* (Balke, 1998), setae are not shown **13** median lobe in ventral view, PNG, Morobe **A**, **B** Kobau **C** Yakob, PNG74 **D** Lae-Bulolo, paratype of *Exocelina patepensis* (Balke, 1998) **14** median lobe in lateral view, IN **A** West Papua, Wasior **B** Papua, Nabire-Ilaga, 96#13 **C** Papua, Wano, Pap027 **D** Papua, Bime-Calab, 16, paratype of *Exocelina rivulus* (Balke, 1998).

**Figure 15.**
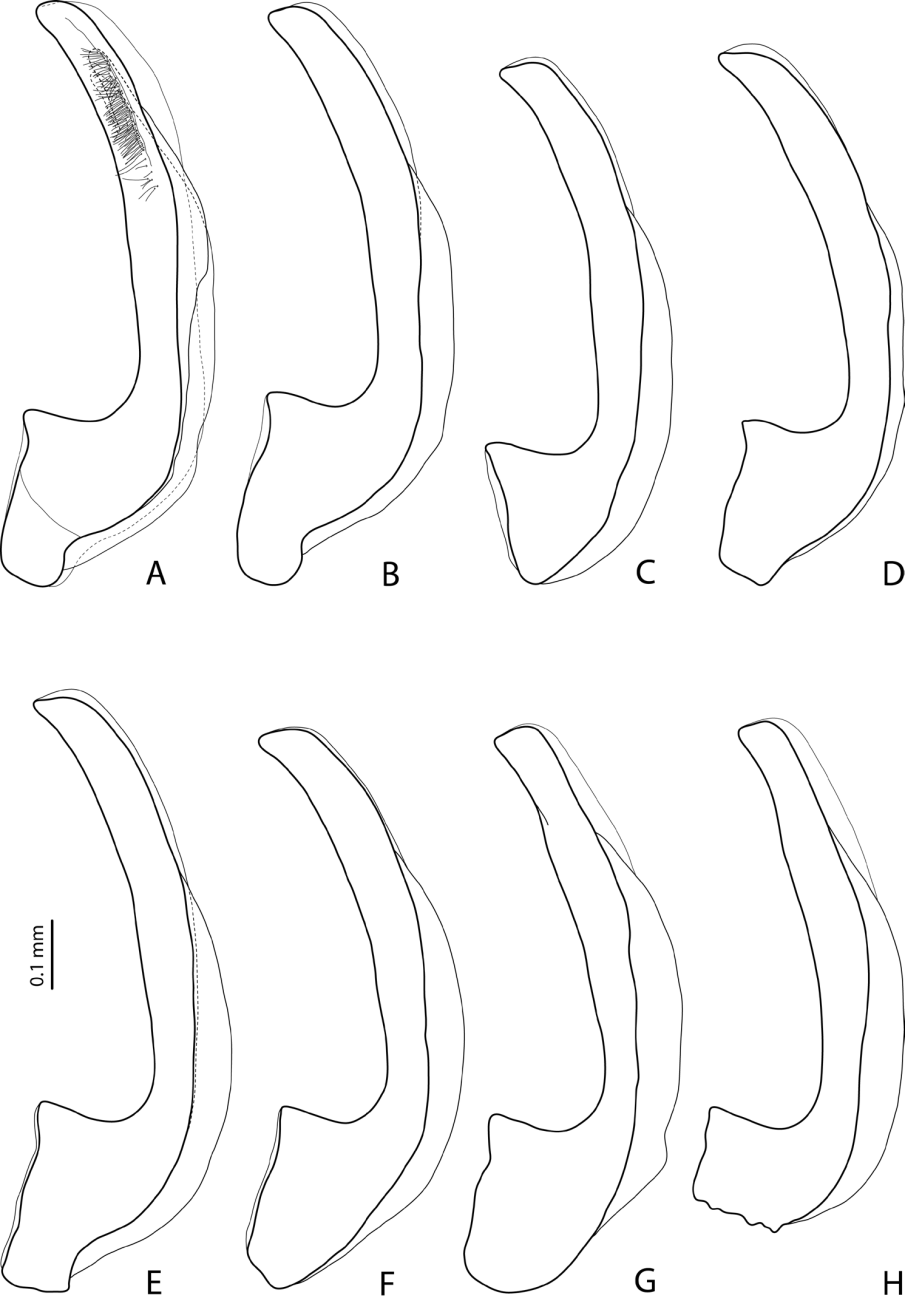
*Exocelina damantiensis* (Balke, 1998), median lobe in lateral view, setae are not shown **A** IN, Papua, Angguruk, 32, paratype of *Exocelina rivulus* **B** PNG, Sandaun, Mianmin area, PNG236 **C**, **D** PNG, Western Province, Tabubil, PNG181 **E** PNG, Enga, PNG128 **F** PNG, WHL, PNG147 **G** PNG, Simbu/EHL, Hulene River, PNG17 **H** PNG, Simbu/EHL, Wara Sera, PNG10.

**Figure 16.**
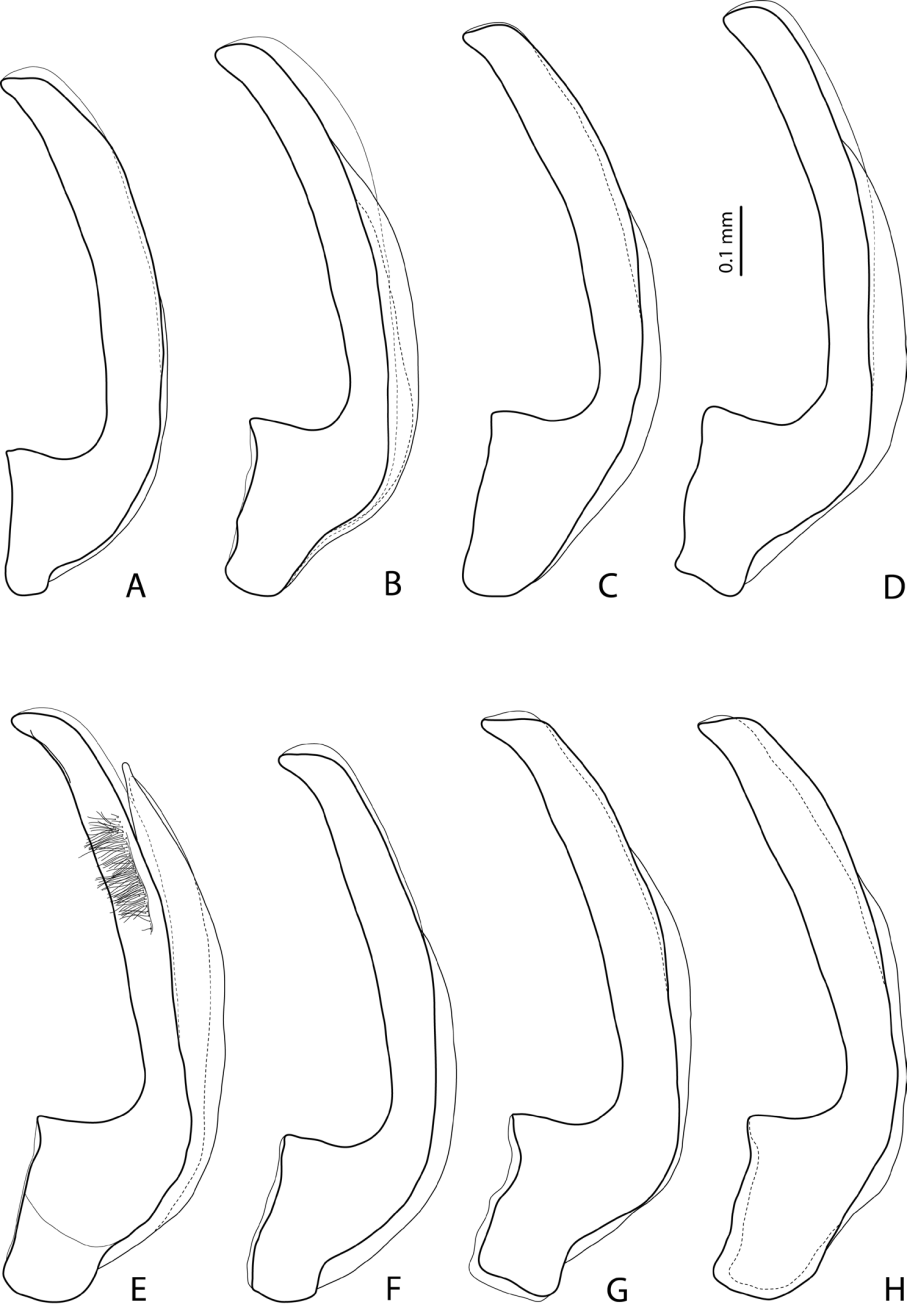
*Exocelina damantiensis* (Balke, 1998), PNG, median lobe in lateral view, setae are not shown **A** Madang, Brahman-Bundi, paratype of *Exocelina madangensis* (Balke, 2001) **B** Madang, Akameku-Brahman, PNG114 **C** Madang, Damanti, paratype of *Exocelina damantiensis* **D** Morobe, Yakob, PNG74 **E** Morobe, Lae-Bulolo, paratype of *Exocelina patepensis* (Balke, 1998) **F** Morobe, Kwapsanek, PNG25 **G**, **H** Morobe, Kobau.

Herein, we synonymize with *Exocelina damantiensis* three species, which were described and treated as representatives of the *Exocelina rivulus*-group: *Exocelina madangensis*, *Exocelina patepensis*, and *Exocelina rivulus* (Balke 1998). The main difference between these species was in the shape of the medial lobe, therefore, this character was carefully studied in all available populations and illustrated (in ventral and lateral views) for almost all of them (Figs 10–16). It has been found that the shape of the median lobe varies both within and among populations. It can be slightly (e.g., Figs 10B, D, 11A, C, F, 12D, E, F, 13C) or strongly (e.g., Figs 10A, 12A–C, H, 13A, B, D) broadened subdistally. Sometimes, the narrower shape might be due to the fact that specimens are teneral (e.g., Fig. 12D) or were treated for SEM (e.g., paratype of *Exocelina madangensis*, Fig. 12E). The shape can be less and more narrowed apically, the narrower form being characteristic of eastern populations: Simbu, EHL, Madang, and Morobe, but is also found in specimens from Papua, Sandaun, and the Western Province. The less narrowed apically shape is characteristic of specimens from one population in Morobe (Yakop, Fig. 13C), and sometimes both shapes are found in the same population (Tabubil, Western Pr., Fig. 11E, F). The males of the type series of *Exocelina patepensis* from the Lae–Bulolo region (Morobe) have a median lobe with a more elongate, almost truncate apex (Figs 13D, 16E), but a similar shape can be also observed in some specimens from the other Morobe populations or in some paratypes of *Exocelina damantiensis* from the Finisterre Range, Madang. A less elongate apex of the median lobe is found in the population from the border region Simbu/EHL (Fig. 15G, H). In short, the shape of the median lobe is not a reliable character to support the earlier recognized species, or to split the present material into several new species or subspecies. The other characters, such as size, coloration, and dorsal punctation, vary little between localities. The beetles are medium-sized, piceous or reddish brown (probably more teneral forms), often with a paler head and pronotal sides, sometimes with reddish sutural lines on the elytra, and are shiny dorsally, with the punctation on the elytra fine, rather distinct or almost invisible. Protarsomere 4 always has a large, thick, strongly curved anterolateral hook-like seta; indication of the “small antero-lateral hook” for *Exocelina madangensis* in Balke (1998) is probably a mistake.

Thus, the *Exocelina rivulus*-group is apparently not a complex of several species, but instead a single, very successful polymorphic species *Exocelina damantiensis*, with the largest geographical range of any New Guinea *Exocelina*: along the central mountain range from Wandammen Peninsula to Huon Peninsula. Of course, it is not out of the question that further material and study of other aspects (e.g., population genomics) might change this situation.

#### Distribution and habitats.

Indonesia and PNG. It occurs in the central mountain chain and the mountains of Wandammen and Huon Peninsulas (Fig. 39). There, it is one of the most common and numerous species from 450 m to 1900 m. Usually, it is a dominate species in the biotope and co-occurs with many species, some of which are mentioned under “Females of doubtful identity”.

### 7. Exocelina danae

Taxon classificationAnimaliaColeopteraDytiscidae

(Balke, 1998)

Figs 19 , 32

Copelatus (Papuadytes) danae Balke, 1998: 328; Nilsson 2001: 76 (catalogue).
Papuadytes danae (Balke, 1998): Nilsson and Fery 2006: 56 (comb. n.).
Exocelina danae (Balke, 1998): Nilsson 2007: 33 (comb. n.).
Copelatus (Papuadytes) tarmluensis Balke, 1998: 338; Nilsson 2001: 77 (catalogue); **syn.n.**
Papuadytes tarmluensis (Balke, 1998): Nilsson and Fery 2006: 56 (comb. n.).
Exocelina tarmluensis (Balke, 1998): Nilsson 2007: 34 (comb. n.).
Exocelina undescribed sp. MB0673: Toussaint et al. 2014: supplementary figs 1–4, tab. 2.

#### Type locality.

Indonesia: Papua Province: Pegunungan Bintang Regency, Aipomek area, between Bime and Tanime, 04°27'S; 140°06'E, 1600 m a.s.l.

#### Type material studied.

***Exocelina danae***: *Holotype*: male “IRIAN JAYA Aipomek area 140°06'E 04°27'S”, “21.8.1992, 1600m, Bime - Tanime leg. Balke (18)”, “HOLOTYPUS” [red], “Copelatus danae Balke des. 1997” [red] (NHMW). *Paratypes*: 5 males, 3 females with the same label as the holotype and additionally with red labels “Paratypus Copelatus danae Balke des. 1997” (NHMW). ***Exocelina tarmluensis***: *Holotype*: male “IRIAN JAYA: Borma, Tarmlu, 1500m, 6.9.1993”, “ca. 140°25'E 04°24'S leg. Balke (4-6)”, “HOLOTYPUS” [red], “Copelatus tarmluensis Balke des. 1997” [red] (NHMW). *Paratypes*: 1 male with the same label as the holotype (NHMW). 1 male “IRIAN JAYA: Borma, Tarmlu, 1500m, 6.9.1993”, “ca. 140°25'E 04°24'S leg. Balke (5)” (NHMW). 2 males “IRIAN JAYA: Borma, Tarmlu, 1500m, 6.9.1993”, “ca. 140°25'E 04°24'S leg. Balke (4)” (NHMW). All paratypes are additionally with red labels “Paratypus Copelatus tarmluensis Balke des. 1997”.

#### Additional material.

**PNG: Sandaun**: 1 male “Papua New Guinea: Sandaun, Sokamin4, 1200m, 19.x.2003, 4 50.845S 141 37.865E, K. Sagata (WB 102)” (ZSM). 1 male “DNA M. Balke 673”, “Papua New Guinea: Sandaun, Mianminold [sic!], 898m, 20.x.2003, 4 53.419S 141 37.028E, K. Sagata (WB66)” (ZSM).

#### Diagnosis.

Beetle medium-sized (TL-H 3.4–4.1 mm); uniformly dark brown to piceous or with paler pronotal sides; shiny, with very fine punctation and microreticulation; pronotum with distinct lateral bead; male antennae simple (Fig. 19D); protarsomere 4 with very small, weakly curved anterolateral “hook-like” (not modified into a hook) seta, smaller than more laterally situated large seta; male protarsomere 5 ventrally with anterior band of more than 40 and posterior row of 7 relatively long, thin setae; median lobe evenly curved, with elongate and broadly pointed apex in lateral view, evenly tapering, with rounded apex in ventral view, on both lateral sides with fine setae situated linearly on anterior half of distal part of median lobe under fine carina; paramere with notch on dorsal side and very dense, strong setae on subdistal part and fine proximal setae (Figs 19A–C, E, F).

**Figures 17–18.**
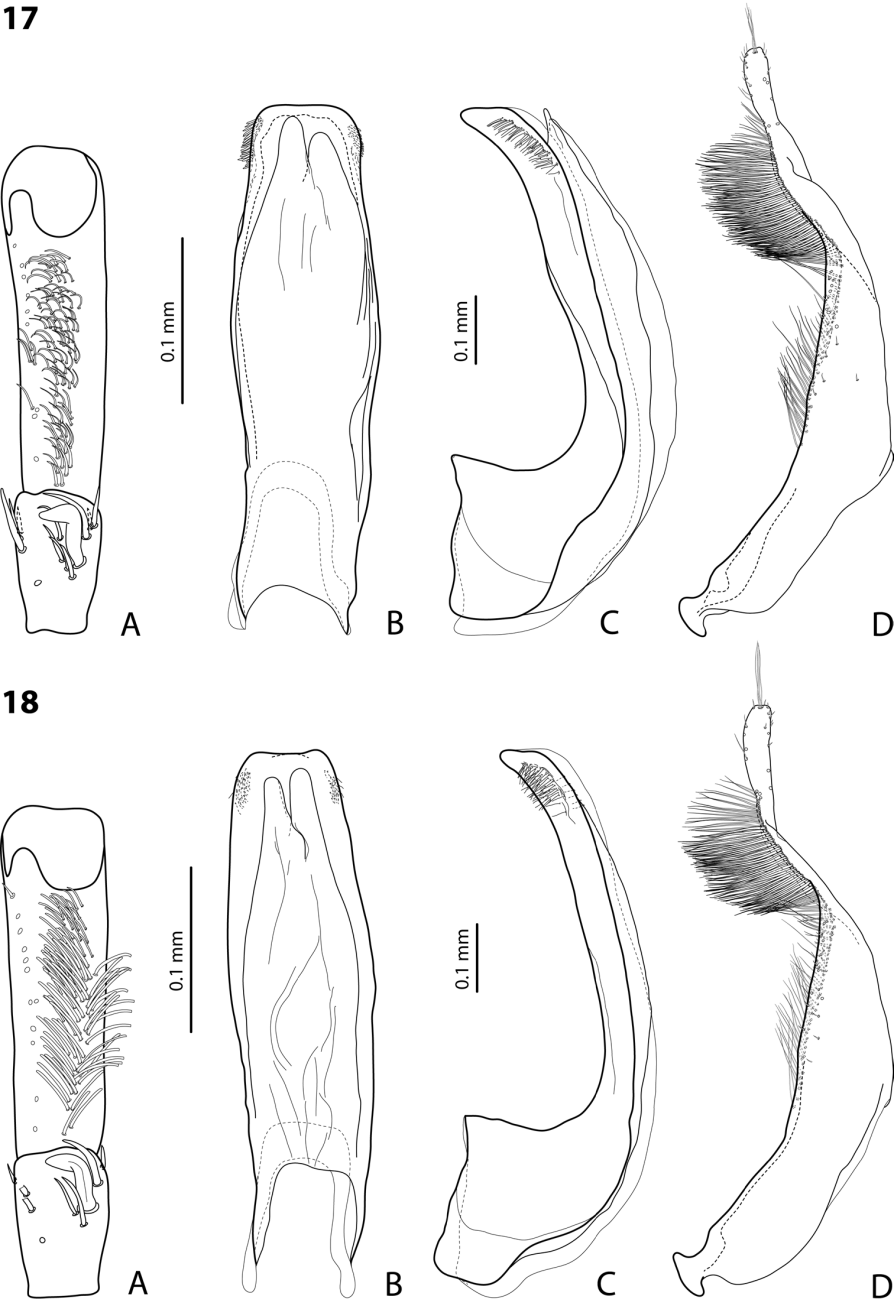
**17** *Exocelina wareaga* sp. n. **18** *Exocelina varirata* sp. n. **A** male protarsomeres 4–5 in ventral view **B** median lobe in ventral view **C** median lobe in lateral view **D** paramere in external view.

**Figure 19.**
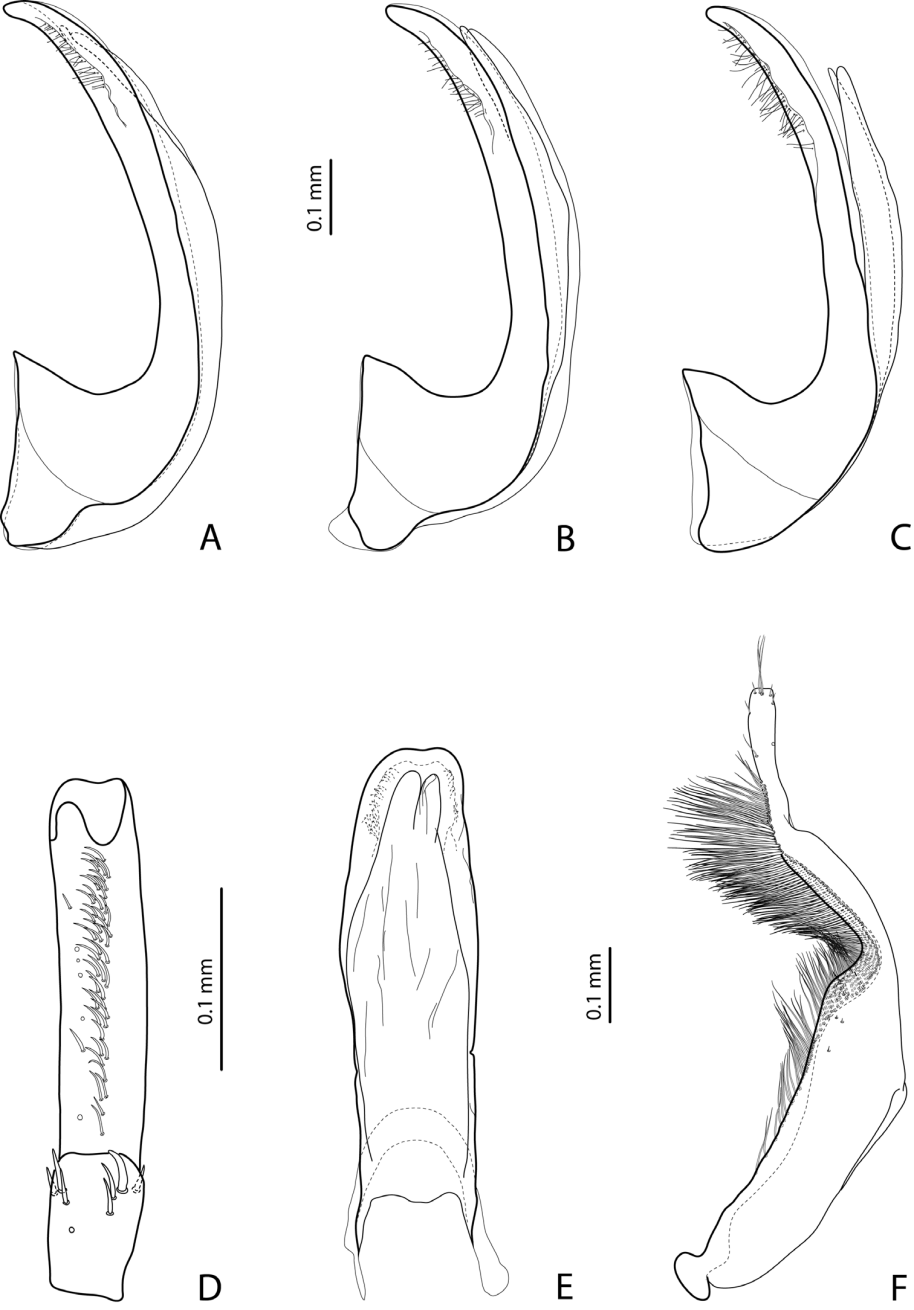
*Exocelina danae* (Balke, 1998) **A–C** median lobe in lateral view **A** paratype of *Exocelina danae* **B** paratype of *Exocelina tarmluensis* (Balke, 1998) **C–F** PNG, Sandaun, Sokamin4, WB102 **D** male protarsomeres 4–5 in ventral view **E** median lobe in ventral view **F** paramere in external view.

Our study of the types of *Exocelina tarmluensis* revealed no significant difference of this species from *Exocelina danae* in the external morphology and in the structure of its genitals. Only slight variability in the shape of the apex of the median lobe was noted (Figs 19A–C). Therefore, *Exocelina tarmluensis* is recognized as a synonym of *Exocelina danae*.

#### Distribution.

Indonesia: Papua Province: Pegunungan Bintang Regency; PNG: Sandaun Province (Fig. 40).

### 8. Exocelina garana

Taxon classificationAnimaliaColeopteraDytiscidae

Shaverdo & Balke sp. n.

http://zoobank.org/E8822E47-3948-4B40-9032-169007EA9561

Figs 21 , 37

Exocelina undescribed sp. MB3876: Toussaint et al. 2014: supplementary figs 1–4, tab. 2.

#### Type locality.

Papua New Guinea: Morobe Province, Garaina, 07°45'05.8"S; 147°08'57.0"E, 720 m a.s.l.

**Type material.** *Holotype*: male “Papua New Guinea: Garaina, 720m, vi.2008, 07.51.032S 147.07.007E Ibalim & Sosanika PNG216”, “DNA M.Balke 3876”, (ZSM). *Paratype*: 1 female with the same geographical label as the holotype (ZSM).

#### Diagnosis.

Beetle medium-sized, dark brown to piceous; dorsal punctation and microreticulation fine; pronotum with lateral bead; male antennomeres simple; male protarsomere 4 with large, thick, strongly curved anterolateral hook-like seta; median lobe slightly broadened and almost rounded distally, with apex weakly concave in ventral view and evenly curved in lateral view, with numerous fine laterodistal setae; paramere without notch on dorsal side; subdistal setae dense, proximal inconspicuous. This species is very similar to *Exocelina damantiensis* but differs from it in the shape of the median lobe: almost rounded distally in ventral view and evenly tapering in lateral view; its apex not curved in lateral view, as well in less numerous subdistal setae of the paramere. These morphological characters and the fact that this species is phylogenetically quite isolated from *Exocelina damantiensis* (Toussaint et al. 2014) support its delimitation.

#### Description.

*Size and shape*: Beetle medium-sized (TL-H 4.25–4.5 mm, TL 4.75–5.0 mm, MW 2.2–2.35 mm), with oblong-oval habitus, broadest at elytral middle. *Coloration*: Male distinctly darker than female. Head reddish-brown to almost piceous; pronotum brown to piceous, with paler (reddish to dark brown) sides and darker (piceous) disc; elytron uniformly piceous or dark brown with reddish sutural line; head appendages yellowish-red, legs reddish-brown (Fig. 37).

*Surface sculpture*: Head with dense punctation (spaces between punctures 1–3 times size of punctures), evidently finer and sparser anteriorly; diameter of punctures smaller than diameter of cells of microreticulation. Pronotum with finer, sparser, and more evenly distributed punctation than on head. Elytra with very sparse and fine punctation, almost invisible. Pronotum and elytra with weakly impressed microreticulation, dorsal surface, thus, shiny. Head with microreticulation stronger. Metaventrite and metacoxa distinctly microreticulate, metacoxal plates with longitudinal strioles and transverse wrinkles. Abdominal sternites with distinct microreticulation, strioles, and fine sparse punctation, coarser and denser on two last abdominal sternites.

*Structures*: Pronotum with distinct lateral bead. Base of prosternum and neck of prosternal process with distinct ridge, smooth and not rounded anteriorly, without anterolateral extensions. Blade of prosternal process lanceolate, relatively narrow, convex, with distinct bead and few setae; neck and blade of prosternal process evenly jointed. Abdominal ventrite 6 slightly truncate apically.

*Male*: Antenna simple. Protarsomere 4 with large, thick, strongly curved anterolateral hook-like seta. Protarsomere 5 ventrally with anterior band of ca. 50 and posterior row of 11 relatively long setae (Fig. 21B). Abdominal ventrite 6 with 4 lateral strioles on each side. Median lobe slightly broadened and almost rounded distally, with apex weakly concave in ventral view and evenly tapering in lateral view; on both lateral sides with fine setae situated on distal part of median lobe (Fig. 21B–C). Paramere without notch on dorsal side and with dense setae on subdistal part; proximal setae more numerous but inconspicuous (Fig. 21D).

**Figures 20–21.**
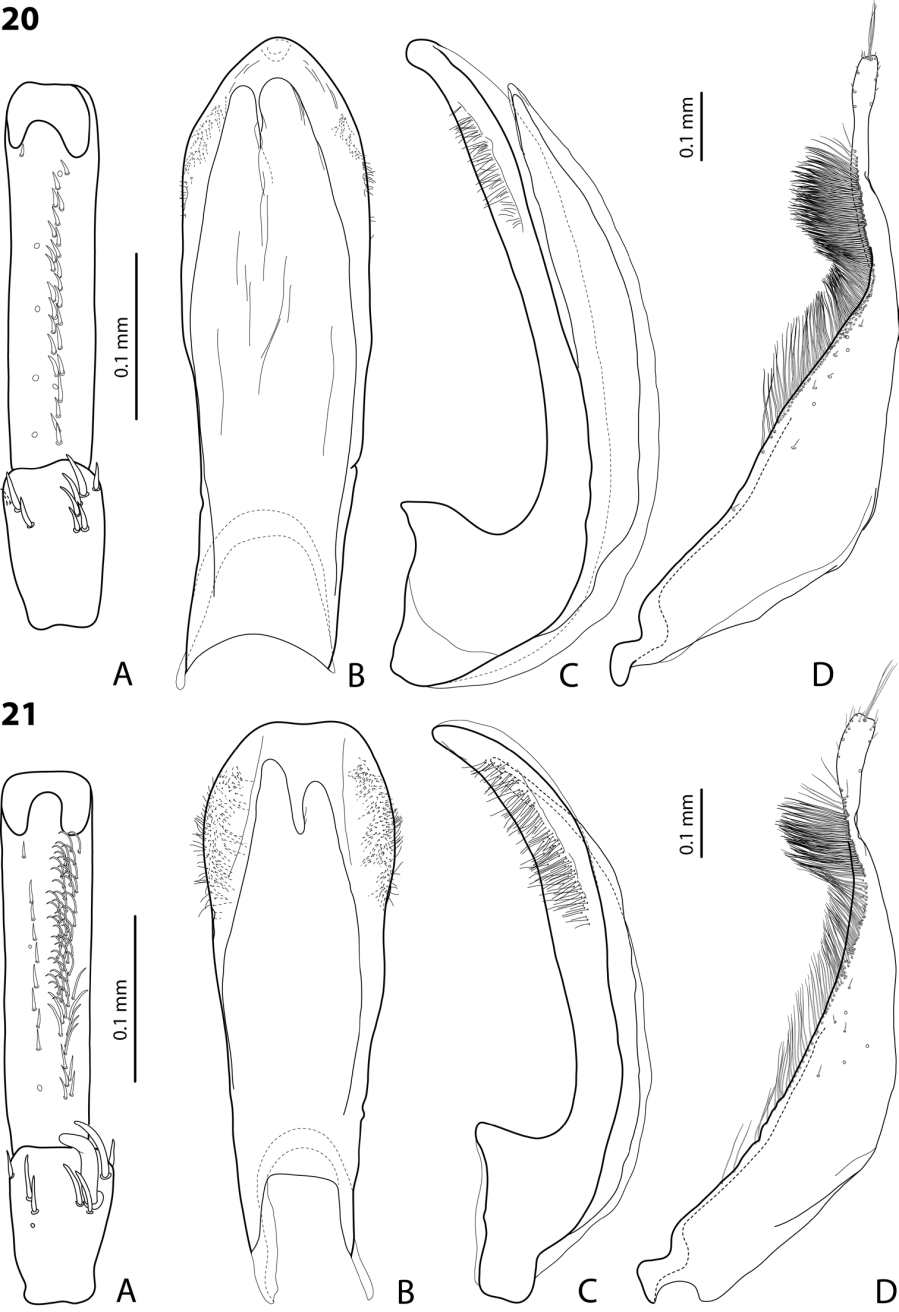
**20** *Exocelina marawaka* sp. n. **21** *Exocelina garaina* sp. n. **A** male protarsomeres 4–5 in ventral view **B** median lobe in ventral view **C** median lobe in lateral view **D** paramere in external view.

*Holotype*: TL-H 4.25 mm, TL 4.75 mm, MW 2.2 mm; dorsally piceous.

*Female*: Pro- and mesotarsi not modified; abdominal ventrite 6 without striae; dorsal coloration paler than in male: dark brown with reddish brown head, pronotal sides and sutural lines on elytra; dorsal punctation slightly stronger.

#### Distribution.

Papua New Guinea: Morobe Province. This species is known only from the type locality area (Fig. 40).

#### Etymology.

The species is named after Garaina Village. The name is a noun in the nominative singular standing in apposition.

### 9. Exocelina injiensis

Taxon classificationAnimaliaColeopteraDytiscidae

Shaverdo & Balke sp. n.

http://zoobank.org/96D815A9-1FE6-4B24-81B3-28FA6BCF38DB

Figs 8 , 30

Exocelina undescribed sp. MB1376: Toussaint et al. 2014: supplementary figs 1–4, tab. 2.

#### Type locality.

Papua New Guinea: Morobe Province, Menyamya, Inji Mountain, 07°14.26'S; 146°01.40'E, 1500 m a.s.l.

**Type material.** *Holotype*: male “Papua New Guinea: Morobe, Menyamya, Mt Inji, deep well, 1500m, 14.xi.2006, 07.14.264S 146.01.400E, Balke & Kinibel (PNG 98)” (ZSM). *Paratypes*: 22 males, 29 females with the same label as the holotype, one male additionally with a green label “DNA M.Balke 1376” (NHMW, ZSM).

#### Diagnosis.

Beetle small; piceous, with reddish brown to brown head and pronotum laterally; matt, with strong punctation and microreticulation; male antennae simple; male protarsomere 4 with large, thick, strongly curved anterolateral hook-like seta; median lobe with slightly curved, rounded apex in lateral view and with almost truncate apex in ventral view, on both lateral sides with strong, short setae situated almost linearly on anterior half of distal part of median lobe under fine carina; paramere without notch on dorsal side. The species is very similar to *Exocelina andakombensis* sp. n. but differs from it in presence of the lateral carina, bordering shorter distal setae, on the median lobe and the large, thick, strongly curved anterolateral hook-like seta of protarsomere 4, as well as longer and much numerous ventral setae of protarsomere 5, see also under diagnosis of *Exocelina andakombensis* sp. n.

#### Description.

*Size and shape*: Beetle small (TL-H 3.05–3.55 mm, TL 3.4–3.85 mm, MW 1.6–1.9 mm), with oblong-oval habitus, broadest at elytral middle. *Coloration*: Head reddish brown to dark brown, with small darker areas posterior to eyes; pronotum reddish brown to dark brown, paler laterally, often piceous on disc; elytra piceous, dark brown laterally, with narrow reddish sutural lines; head appendages and legs proximally yellowish red, legs distally darker, reddish brown, especially metathoracic legs (Fig. 30). Teneral specimens paler.

**Figures 22–23.**
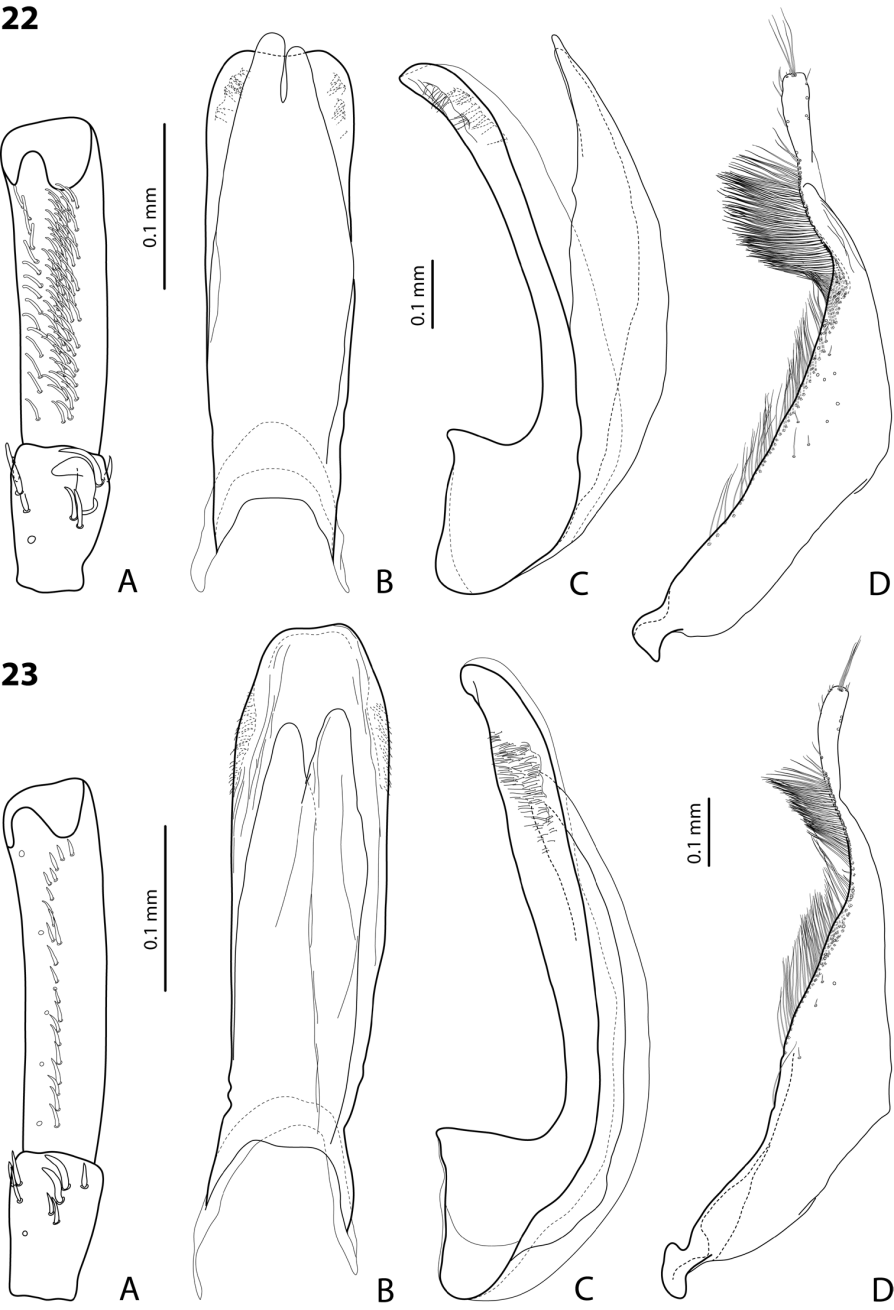
**22** *Exocelina atrata* (Balfour-Browne, 1939) **23** *Exocelina posmani* sp. n. **A** male protarsomeres 4–5 in ventral view **B** median lobe in ventral view **C** median lobe in lateral view **D** paramere in external view.

**Figures 24–26.**
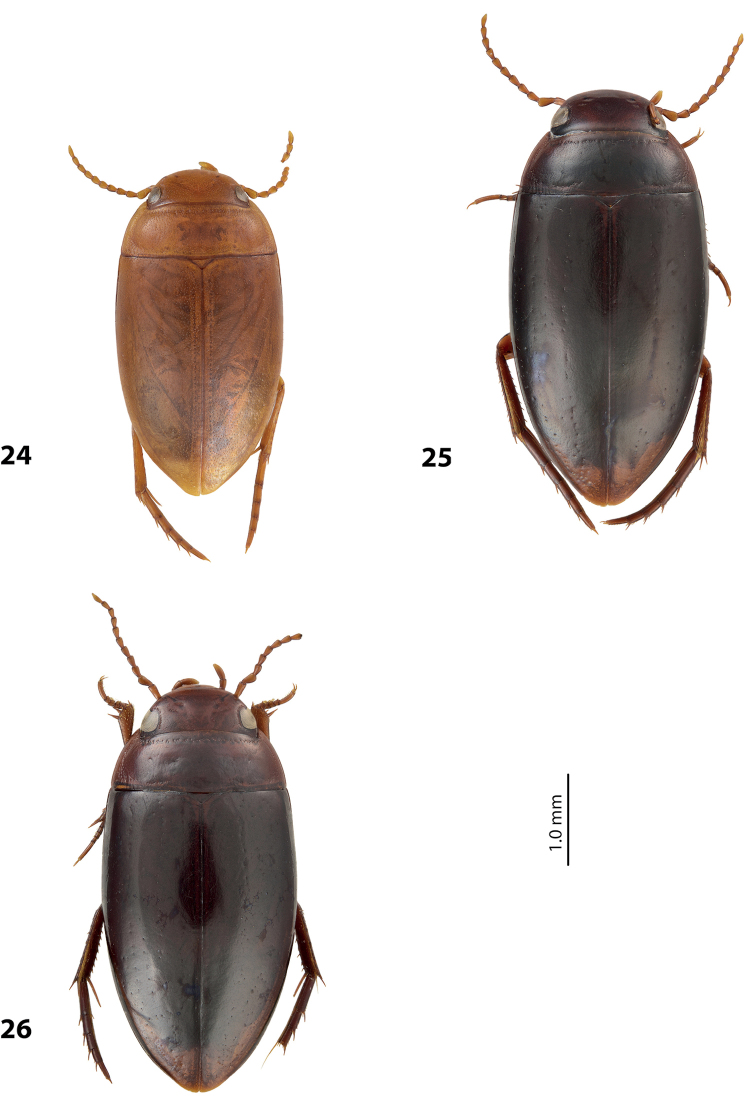
Habitus and coloration **24** *Exocelina rufa* (Balke, 1998) **25** *Exocelina miriae* (Balke, 1998) **26** *Exocelina tekadu* sp. n.

**Figures 27–30.**
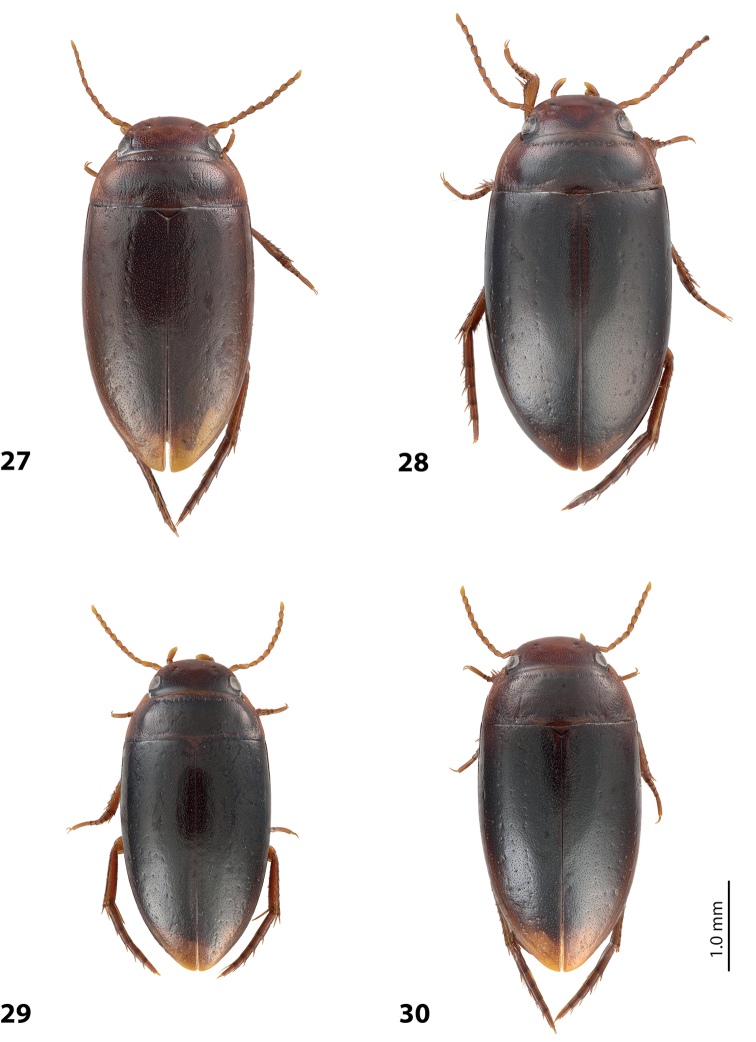
Habitus and coloration **27** *Exocelina kabwumensis* sp. n. **28** *Exocelina woitapensis* sp. n. **29** *Exocelina andakombensis* sp. n. **30** *Exocelina injiensis* sp. n.

*Surface sculpture*: as in *Exocelina andakombensis* sp. n.

*Structures*: Pronotum with distinct lateral bead. Base of prosternum and neck of prosternal process with distinct ridge, slightly rounded anteriorly. Blade of prosternal process lanceolate, relatively broad, slightly convex, and smooth, with distinct lateral bead and few lateral setae; neck and blade of prosternal process evenly jointed. Abdominal ventrite 6 slightly truncate.

*Male*: Antennae simple. Protarsomere 4 with large, thick, strongly curved anterolateral hook-like seta. Protarsomere 5 ventrally with anterior band of 19 and posterior row of 8 relatively long setae (Fig. 8A). Median lobe with slightly curved, rounded apex in lateral view and with almost truncate apex in ventral view, on both lateral sides with strong, short setae situated almost linearly on a half of distal part of median lobe under fine carina (Fig. 8B–C). Paramere without notch, slightly concave on dorsal side and with dense setae on subdistal part; proximal setae inconspicuous (Fig. D). Abdominal ventrite 6 with 6–9 lateral striae on each side.

*Holotype*: TL-H 3.6 mm, TL 3.9 mm, MW 1.9 mm.

*Female*: Without evident differences in external morphology from males, except for not modified pro- and mesotarsi and abdominal ventrite 6 without striae.

#### Distribution.

Papua New Guinea: Morobe Province. The species is known only from the type locality (Fig. 40).

#### Etymology.

The species is named after Inji Mountain. The name is an adjective in the nominative singular.

### 10. Exocelina kabwumensis

Taxon classificationAnimaliaColeopteraDytiscidae

Shaverdo & Balke sp. n.

http://zoobank.org/CBA1FBC1-3873-4046-987D-F9D6F28CC0A1

Figs 5 , 27

Exocelina undescribed sp. MB1285: Toussaint et al. 2014: supplementary figs 1–4, tab. 2.

#### Type locality.

Papua New Guinea: Morobe, Huon, Kabwum, 06°08.01'S; 147°11.34'E, 1600 m a.s.l.

#### Type material.

*Holotype*: male “Papua New Guinea: Morobe, Huon, 1 km SE Kabwum, 1600m, 16./17.v.2006, 06.08.007S 147.11.337E, Sagata (PNG 76)”, “DNA M.Balke 1285” [green] (ZSM). *Paratypes*: 3 males, 3 females with the same labels as the holotype (NHMW, ZSM).

#### Diagnosis.

Beetle small to medium-sized; piceous, with reddish brown to brown head and pronotum laterally; matt, with strong punctation and microreticulation; male antennae simple; protarsomere 4 with weakly curved anterolateral “hook-like” (not modified into a hook) seta, smaller than more laterally situated large seta; median lobe narrow, with almost parallel sides and askew truncate apex in ventral view, with slightly curved apex and very few fine distal setae in lateral view; paramere with small notch on dorsal side. The species is very similar to *Exocelina andakombensis* sp. n., *Exocelina injiensis* sp. n., and *Exocelina woitapensis* sp. n., but differs from them in size, shape of the median lobe, and the presence of only few fine distal setae laterally on the median lobe.

#### Description.

*Size and shape*: Beetle small to medium-sized (TL-H 3.5–3.8 mm, TL 3.75–4.15 mm, MW 1.85–2.05 mm), with oblong-oval habitus, broadest at elytral middle. *Coloration*: as in *Exocelina andakombensis* sp. n. (Fig. 27).

*Surface sculpture*: As in *Exocelina andakombensis* sp. n.

*Structures*: Pronotum with distinct lateral bead. Base of prosternum and neck of prosternal process with distinct ridge, slightly rounded anteriorly. Blade of prosternal process lanceolate, relatively broad, slightly convex, and smooth, with distinct lateral bead and few lateral setae; neck and blade of prosternal process evenly jointed. Abdominal ventrite 6 slightly truncate or broadly rounded.

*Male*: Antennae simple. Protarsomere 4 with very small, weakly curved anterolateral “hook-like” (not modified into a hook) seta, smaller than more laterally situated large seta. Protarsomere 5 ventrally with anterior band of 27 and posterior row of 6 relatively long, thin setae (Fig. 5A). Median lobe narrow, with almost parallel sides and askew truncate apex in ventral view, with slightly curved, relatively broad apex and very few fine distal setae in lateral view (Figs 5B–C). Paramere with small notch on dorsal side and with dense setae on subdistal part; proximal setae inconspicuous (Fig. 5D). Abdominal ventrite 6 with 8–10 lateral striae on each side.

*Holotype*: TL-H 3.65 mm, TL 4.0 mm, MW 2.0 mm.

*Female*: Without evident differences in external morphology from males, except for not modified pro- and mesotarsi and abdominal ventrite 6 without striae.

#### Distribution.

Papua New Guinea: Morobe Province. The species is known only from the type locality (Fig. 40).

#### Etymology.

The species is named after Kabwum Village. The name is an adjective in the nominative singular.

### 11. Exocelina marawaka

Taxon classificationAnimaliaColeopteraDytiscidae

Shaverdo & Balke sp. n.

http://zoobank.org/421FB9C2-1F26-4764-AE88-33CB5E9C9E84

Figs 20 , 35

Exocelina undescribed sp. MB1366: Toussaint et al. 2014: supplementary figs 1–4, tab. 2.

#### Type locality.

Papua New Guinea: Eastern Highlands Province, Marawaka, Ande, 07°01.70'S; 145°49.81'E, 1700 m a.s.l.

#### Type material.

*Holotype*: male “Papua New Guinea: Eastern Highlands, Marawaka, Ande, 1700m, 8.xi.2005, 07.01.697S 145.49.807E, Balke & Kinibel (PNG 86)” (ZSM). *Paratypes*: **Eastern Highlands**: 32 males, 17 females with the same label as the holotype, one male with a green label “DNA M.Balke 1366” (NHMW, ZSM). 8 males, 6 females “Papua New Guinea: Eastern Highlands, Marawaka, Ande, 1700-1800m, 9.xi.2006, 07.01.697S 145.49.807E, Balke & Kinibel (PNG 87)” (NHMW, ZSM). **Gulf**: 1 female “Papua New Guinea: Gulf, Marawaka, Andakombe towards Morobe, 2160m, 12.xi.200, 07.11.717S 145.51.177E, Balke & Kinibel (PNG 94)”, “DNA M.Balke 1370” [green] (ZSM).

#### Diagnosis.

Beetle medium-sized, piceous, with paler sides of pronotum; dorsal surface with fine punctation and evident microreticulation, shiny; pronotum with distinct lateral bead; male antennomeres simple; protarsomere 4 with weakly curved anterolateral hook-like seta, smaller than more laterally situated large seta; median lobe evidently broadened in distal part, broadly pointed to apex in ventral view and with slightly curved, rounded apex in lateral view, on both lateral sides with numerous fine setae situated linearly on anterior half of distal part of median lobe under fine carina; paramere without notch on dorsal side. The species is similar to *Exocelina posmani* sp. n. but differs from it mainly in the structure of the median lobe: apex longer and narrower in lateral view and pointed in ventral view, distal setae not arranged into one area but situated linearly along the lateral margin.

#### Description.

*Size and shape*: Beetle medium-sized (TL-H 4.05–4.6 mm, TL 4.4–5.0 mm, MW 2.15–2.45 mm), with oblong-oval habitus, broadest at elytral middle. *Coloration*: Head uniformly dark brown to piceous; pronotum dark brown to piceous, paler on sides; elytra uniformly piceous; ventrally dark brown; head appendages and legs proximally yellowish red, legs distally darker, reddish brown (Fig. 35).

**Figures 31–34.**
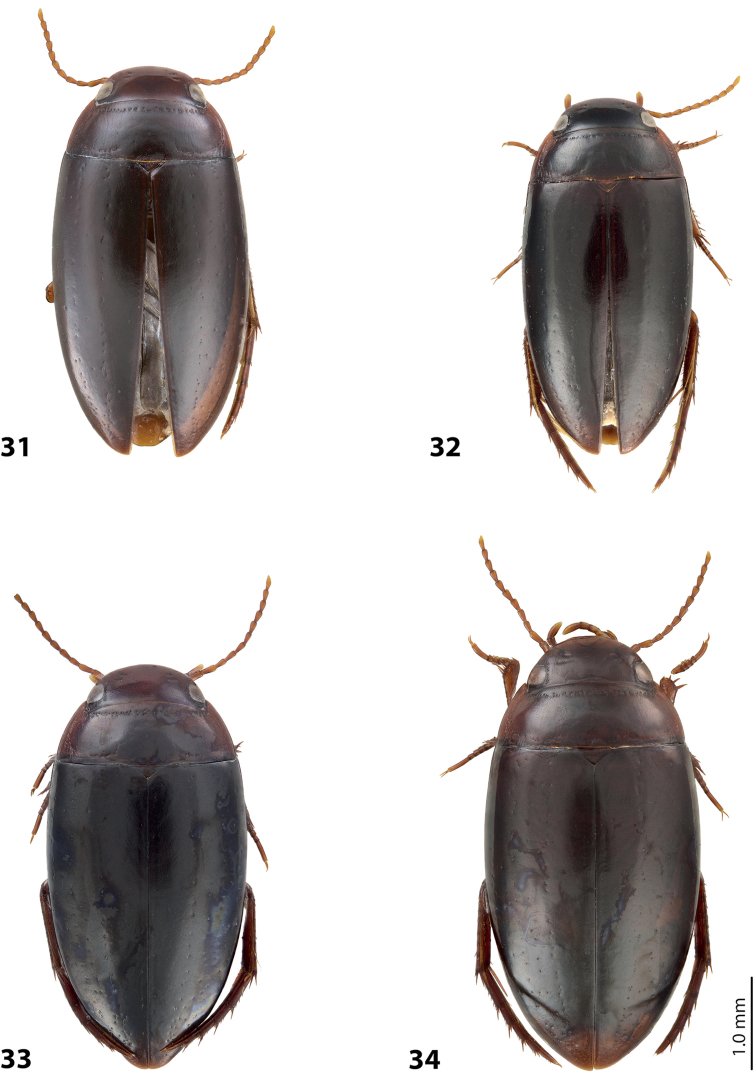
Habitus and coloration **31** *Exocelina damantiensis* (Balke, 1998) **32** *Exocelina danae* (Balke, 1998) **33** *Exocelina wareaga* sp. n. **34** *Exocelina varirata* sp. n.

**Figures 35–38.**
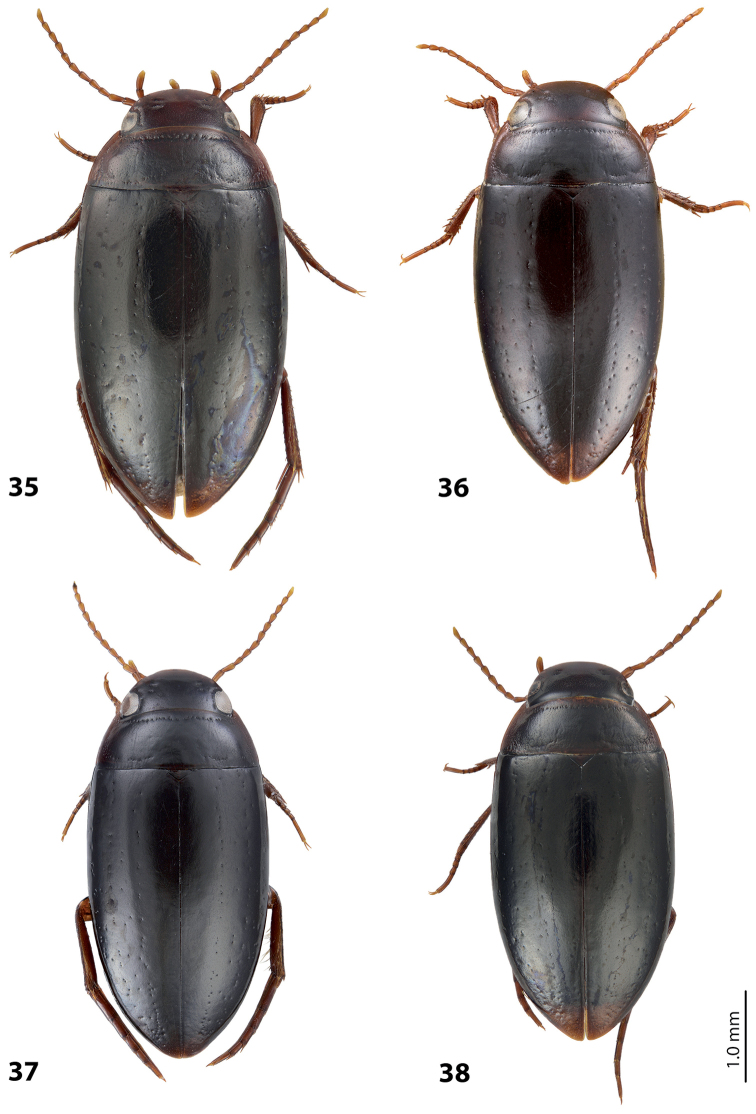
Habitus and coloration **35** *Exocelina marawaka* sp. n. **36** *Exocelina atrata* (Balfour-Browne, 1939) **37** *Exocelina garaina* sp. n. **38** *Exocelina posmani* sp. n.

*Surface sculpture*: Head with relatively dense and coarse punctation (spaces between punctures 1–3 times size of punctures); diameter of punctures smaller than diameter of cells of microreticulation. Pronotum with finer, sparser, and more evenly distributed punctation than on head. Elytra with much finer, sparser punctation than on pronotum. Pronotum and elytra with distinct microreticulation, dorsal surface shiny. Head with microreticulation slightly stronger. Metaventrite, metacoxa, and abdominal ventrites distinctly microreticulate. Metacoxal plates with longitudinal strioles and transverse wrinkles; abdominal ventrites with strioles. Ventrum with inconspicuous punctation, more evident on metacoxal plates and two last abdominal ventrites.

*Structures*: Pronotum with distinct lateral bead. Base of prosternum and neck of prosternal process with distinct ridge, not rounded anteriorly, without anterolateral extensions. Blade of prosternal process lanceolate, relatively narrow, convex, with distinct bead and few setae laterally; neck and blade of prosternal process evenly jointed. Abdominal ventrite 6 broadly rounded or slightly truncate.

*Male*: Antenna simple. Protarsomere 4 with very small (smaller than more laterally situated large seta), weakly curved anterolateral hook-like seta. Protarsomere 5 ventrally with anterior band of 27 setae and posterior row of 5 short, relative thick setae (Fig. 20A). Abdominal ventrite 6 with 7–10 lateral striae on each side. Median lobe evidently broadened in distal part, braodly pointed to apex in ventral view and with slightly curved, rounded apex in lateral view, on both lateral sides with numerous fine setae situated linearly on anterior half of distal part of median lobe under fine carina (Fig. 20B–C). Paramere without notch, slightly concave on dorsal side, with thin, sparse, inconspicuous proximal setae and thicker, denser, and longer subdistal setae (Fig. 20D).

*Holotype*: TL-H 4.5 mm, TL 4.9 mm, MW 2.2 mm.

*Female*: Without evident differences in external morphology from males, except for not modified pro- and mesotarsi and abdominal ventrite 6 without striae.

#### Variability.

Elytral punctation varies from inconspicuous to distinct.

#### Distribution.

Papua New Guinea: Eastern Highlands and Gulf Provinces. The species is known only from the Marawaka area (Fig. 40).

**Figure 39.**
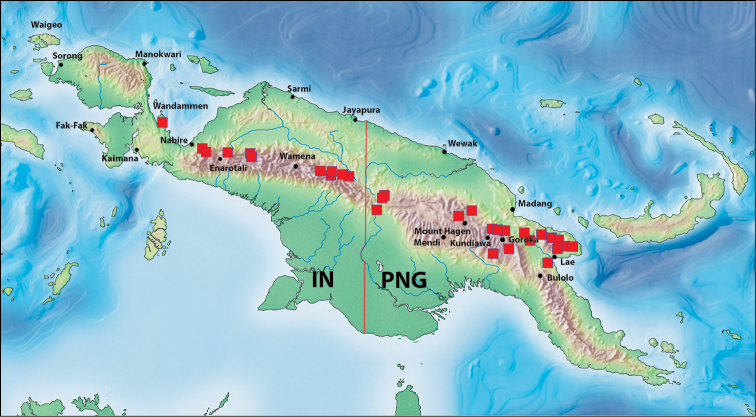
Map of New Guinea showing distribution of *Exocelina damantiensis* (Balke, 1998).

**Figure 40.**
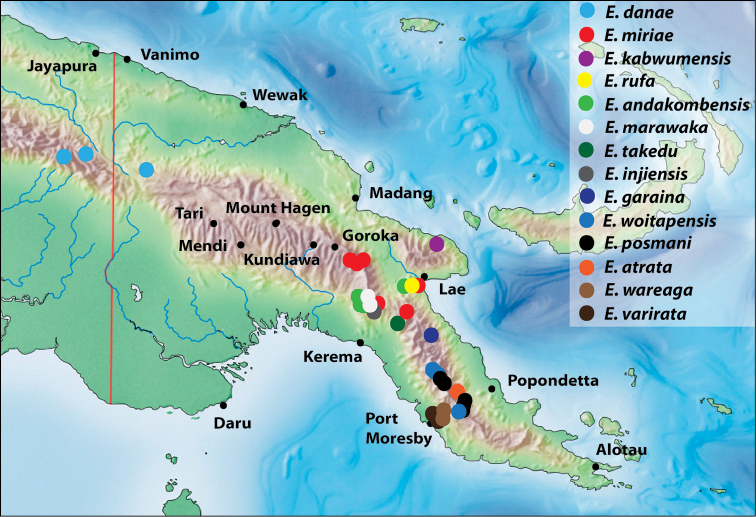
Map of Papua New Guinea showing distribution of species of the *Exocelina danae*-group.

#### Etymology.

The species is named after the Marawaka area. The name is a noun in the nominative singular standing in apposition.

### 12. Exocelina posmani

Taxon classificationAnimaliaColeopteraDytiscidae

Shaverdo & Balke sp. n.

http://zoobank.org/F0F916E9-4088-43E0-B068-3D6DB744833F

Figs 23 , 38

Exocelina undescribed sp. MB3406: Toussaint et al. 2014: supplementary figs 1–4, tab. 2.

#### Type locality.

Papua New Guinea: Central Province, Myola, 09°08.05'S; 147°42.24'E, 1760 m a.s.l.

#### Type material.

*Holotype*: male “Papua New Guinea: Central, Myola, 1760m, i.2008, [09°] 08.052S 147 42.241E, Posman (PNG 176)” (ZSM). *Paratypes*: 2 males, 4 females with the same label as the holotype, one male with an additional green label “DNA M.Balke 3406” (NHMW, ZSM). 1 male, 1 female “Papua New Guinea: Central, Woitape, 1700m, i.2008, [08°] 31.290S 147 13.684'E, Posman (PNG 166)” (ZSM). 2 males “Papua New Guinea: Central, Woitape, 1500m, i.2008, [08°] 33.178S 147 15.481E, Posman (PNG 167)” (NHMW, ZSM). 4 males, 2 females “Papua New Guinea: Central, Kokoda Trek, 1400m, i.2008, [09°] 14.339S 147 40.538E, Posman (PNG 171)” (NHMW, ZSM). 1 female “Papua New Guinea: Central, Kokoda Trek, 1400m, i.2008, [09°] 01.952S 147 44.455E, Posman (PNG 172)” (ZSM).

#### Diagnosis.

Beetle medium-sized, piceous, with paler head and sides of pronotum; dorsal surface with fine punctation and evident microreticulation, shiny; pronotum with distinct lateral bead; male antennomeres simple; protarsomere 4 with weakly curved anterolateral hook-like seta, equal to more laterally situated large seta; median lobe only slightly broadened in distal part, with almost parallel sides and slightly concave apex in ventral view, with slightly curved, broad, rounded apex in lateral view, on both lateral sides with numerous fine setae situated not linearly but on large area of anterior half of distal part of median lobe under short fine carina; paramere without notch on dorsal side. The species is similar to *Exocelina marawaka* sp. n. but differs from it in the structure of the median lobe: apex shorter and broader in lateral view and slightly concave in ventral view, distal setae arranged into one area, not situated linearly. This species was collected together with of *Exocelina woitapensis* sp. n., which is smaller and matt, with stronger punctation and microreticulation of the dorsal surface.

#### Description.

*Size and shape*: Beetle medium-sized (TL-H 3.65–4.4 mm, TL 4–4.5 mm, MW 1.95–2.2 mm), with oblong-oval habitus, broadest at elytral middle. *Coloration*: Head uniformly dark brown to piceous or reddish-brown to brown, dark brown behind eyes and on middle; pronotum dark brown to piceous, paler on sides; elytra uniformly dark brown to piceous, seldom with narrow reddish sutural lines; ventrally reddish-brown; head appendages and legs proximally yellowish red, legs distally darker, reddish brown (Fig. 38).

*Surface sculpture*: As in *Exocelina marawaka* sp. n.

*Structures*: Pronotum with distinct lateral bead. Base of prosternum and neck of prosternal process with distinct ridge, not rounded anteriorly, without anterolateral extensions. Blade of prosternal process lanceolate, relatively narrow, convex, with distinct bead and few setae laterally; neck and blade of prosternal process evenly jointed. Abdominal ventrite 6 broadly rounded.

*Male*: Antenna simple. Protarsomere 4 with small (equal to more laterally situated large seta), weakly curved anterolateral hook-like seta. Protarsomere 5 ventrally with anterior band of 22 setae and posterior row of 5 short setae (Fig. 23A). Abdominal ventrite 6 with 4–7 lateral striae on each side. Median lobe only slightly broadened in distal part, with almost parallel sides and slightly concave apex in ventral view, with slightly curved, broad, rounded apex in lateral view, on both lateral sides with numerous fine setae situated not linearly but on large area of anterior half of distal part of median lobe under short, fine carina (Fig. 23B–C). Paramere without notch, slightly concave on dorsal side, with thin, sparse, inconspicuous proximal setae and thicker, denser, and longer subdistal setae (Fig. 23D).

*Holotype*: TL-H 3.9 mm, TL 4.35 mm, MW 2.15 mm.

*Female*: Without evident differences in external morphology from males, except for not modified pro- and mesotarsi and abdominal ventrite 6 without striae.

#### Variability.

Elytral punctation varies from inconspicuous to distinct.

#### Distribution.

Papua New Guinea: Central Province (Fig. 40).

#### Etymology.

The species is named for Aloysius Posman. The species name is a noun in the genitive case.

### 13. Exocelina varirata

Taxon classificationAnimaliaColeopteraDytiscidae

Shaverdo & Balke sp. n.

http://zoobank.org/610FF2E1-A763-4AF1-AABA-FEA533636427

Figs 18 , 34

Exocelina undescribed sp. MB3303: Toussaint et al. 2014: supplementary figs 1–4, tab. 2.

#### Type locality.

Papua New Guinea: National Capital District Province, Varirata National Park, 09°26.13'S; 147°22.09'E, 600 m a.s.l.

#### Type material.

*Holotype*: male “Papua New Guinea: National Capital District, Varirata NP, 600m, 16.xii.2007, 09.26.13S 147.22.09E, Balke & Sagata (PNG 159)”, “DNA M.Balke 3303” [green] (ZSM). *Paratype*: **Central**: 1 male “Papua New Guinea: Central, Myola, 1110m, i.2008, 09 12.630S 147 31.880E, Posman (PNG 177)”, “DNA M.Balke 3407” [green] (ZSM).

#### Diagnosis.

Beetle medium-sized, dark brown, with reddish-brown pronotal sides; dorsal surface with strong punctation and microreticulation, matt; pronotum with distinct lateral bead; male antennomeres simple; male protarsomere 4 with large, thick, strongly curved anterolateral hook-like seta; median lobe slender, with slightly curved, short, broad apex and compact area of fine distal setae in lateral view, with slightly concave apex in ventral view; paramere with very shallow notch on dorsal side. The species is very similar to *Exocelina wareaga* sp. n. but differs from it in shape of the median lobe: it is more slender, lateral margins apically and subapically not very thick and not bordered with a carina; also the fine distal setae on lateral sides of the median lobe are not situated linearly, but in compact areas.

#### Description.

*Size and shape*: Beetle medium-sized (TL-H 4.25–4.35 mm, TL 4.5–4.85 mm, MW 2.25–2.45 mm), with oblong-oval habitus, broadest at elytral middle. *Coloration*: Head reddish brown to dark brown, paler on clypeus; pronotum dark brown on disc and reddish-brown on sides; elytra uniformly dark brown; ventrally dark brown; head appendages reddish-brown, legs darker distally (Fig. 34).

*Surface sculpture*: as in *Exocelina wareaga* sp. n.

*Structures*: Pronotum with distinct lateral bead. Base of prosternum and neck of prosternal process with distinct ridge, smooth anteriorly, without anterolateral extensions. Blade of prosternal process lanceolate, relatively narrow, convex, with distinct bead and few setae laterally; neck and blade of prosternal process evenly jointed. Abdominal ventrite 6 broadly rounded.

*Male*: Antenna simple. Protarsomere 4 with large, thick, strongly curved anterolateral hook-like seta. Protarsomere 5 ventrally with anterior band of more than 70 short setae and posterior row of 12 relatively long, thin setae (Fig. 18A). Abdominal ventrite 6 with 4–6 lateral striae on each side. Median lobe slender, with slightly curved, short, broad apex and compact area of fine distal setae in lateral view, with slightly concave apex in ventral view (Figs 18B–C). Paramere with very shallow notch on dorsal side, with thin, sparse, inconspicuous proximal setae and thicker, denser, and longer subdistal setae (Fig. 18D).

*Holotype*: TL-H 4.35 mm, TL 4.85 mm, MW 2.45 mm.

*Female*: Unknown.

#### Distribution.

Papua New Guinea: National Capital District and Central Provinces (Fig. 40).

#### Etymology.

The species is named after Varirata National Park. The name is a noun in the nominative singular standing in apposition.

### 14. Exocelina wareaga

Taxon classificationAnimaliaColeopteraDytiscidae

Shaverdo & Balke sp. n.

http://zoobank.org/BC69F25B-00F5-4C97-8854-BBFFB4B008DD

Figs 17 , 33

Exocelina undescribed sp. MB3404: Toussaint et al. 2014: supplementary figs 1–4, tab. 2.

#### Type locality.

Papua New Guinea: Central Province, Moroka, Kailaki, 09°24.13'S; 147°33.52'E, 827 m a.s.l.

#### Type material.

*Holotype*: male “Papua New Guinea Central, Moroka, Kailaki Wareaga, 760m, 27x2009 9.25.424S 147.31.068E Sagata (PNG227)” (ZSM). *Paratypes*: **Central**: 39 males, 46 females with the same label as the holotype (NHMW, ZSM). 7 males, 6 females “Papua New Guinea: Central, Moroka area, Kailaki, 827 m, 26.x.2009, 9.24.134S 147.33.521E, Sagata (PNG225)” (NHMW, ZSM). 10 males, 3 females “Papua New Guinea Central, 755m, 28.x.2009 S9 25 47 5 E147 32 59.1, Sagata (PNG229)” (NHMW, ZSM). 2 males, 2 females “Papua New Guinea: Central, Kokoda Trek, 980m, i.2008, 09 15.933S 147 36.590E, Posman (PNG 169)”, one male and female with green labels “DNA M.Balke 3410” and “DNA M.Balke 4118” correspondently (NHMW, ZSM). 4 males, 6 females “Papua New Guinea: Central, Kokoda Trek, 320m, i.2008 09 19.236S 147 31.791E, Posman (PNG 168)”, one male with a green label “DNA M.Balke 3404” (NHMW, ZSM). 3 males, 2 females “Papua New Guinea: Central, Kokoda Trek, 590m, i.2008, 09 14.339S 147 36.920E, Posman (PNG 170)” (NHMW, ZSM). **National Capital District**: 1 male “Papua New Guinea: National Capital District, Varirata NP, 600m, 16.xii.2007, 09.26.13S 147.22.09E, Balke & Sagata (PNG 159)” [specimen without head and pronotum] (ZSM).

#### Diagnosis.

Beetle medium-sized, dark brown, with paler, reddish-brown, head and pronotum; dorsal surface with fine punctation and evident microreticulation, shiny; pronotum with distinct lateral bead; male antennomeres simple; male protarsomere 4 with large, thick, strongly curved anterolateral hook-like seta; median lobe robust, apicolaterally with thick margins bordered with dorsolateral carina, with slightly curved, broad apex in lateral view and with truncate apex in ventral view, on both lateral sides with numerous fine setae situated linearly on anterior half of distal part of median lobe; paramere with very shallow notch on dorsal side. The species is very similar to *Exocelina varirata* sp. n. but differs from it in the shape of the median lobe: it is more robust, lateral margins apically and subapically thicker, bordered with a dorsolateral carina; also fine distal setae on lateral sides of the median lobe are situated linearly.

#### Description.

*Size and shape*: Beetle medium-sized (TL-H 3.65–4.4 mm, TL 4.05–4.8 mm, MW 1.95–2.35 mm), with oblong-oval habitus, broadest at elytral middle. *Coloration*: Head reddish-brown, dark brown behind eyes; pronotum reddish-brown, dark brown on disc; elytra uniformly brown to dark brown; ventrally reddish-brown, slightly darker on metacoxal plates; head appendages red to reddish-brown, legs darker distally (Fig. 33). Teneral specimens paler, with yellowish-red head and pronotum and pale brown elytra.

*Surface sculpture*: Head with relatively dense and coarse punctation (spaces between punctures 1–3 times size of punctures); diameter of punctures smaller than diameter of cells of microreticulation. Pronotum with finer, sparser, and more evenly distributed punctation than on head. Elytra with finer, sparser punctation than on pronotum, punctation very fine but evident. Pronotum and elytra with distinct microreticulation, dorsal surface shiny. Head with microreticulation slightly stronger. Metaventrite, metacoxa, and abdominal ventrites distinctly microreticulate, but with cells of microreticulation larger than on dorsal side. Metacoxal plates with longitudinal strioles and transverse wrinkles; abdominal ventrites with strioles. Ventrum with inconspicuous punctation, more evident on metacoxal plates and two last abdominal ventrites.

*Structures*: Pronotum with distinct lateral bead. Base of prosternum and neck of prosternal process with distinct ridge, smooth anteriorly, without anterolateral extensions. Blade of prosternal process lanceolate, relatively narrow, convex, with distinct bead and few setae laterally; neck and blade of prosternal process evenly jointed. Abdominal ventrite 6 slightly truncate.

*Male*: Antenna simple. Protarsomere 4 with large, thick, strongly curved anterolateral hook-like seta. Protarsomere 5 ventrally with anterior band of more than 70 short setae and posterior row of 13 relatively long, thin setae (Fig. 17A). Abdominal ventrite 6 with 3–5 lateral striae on each side. Median lobe robust, apicolaterally with thick margins bordered with dorsolateral carina, with slightly curved, broad apex in lateral view and with truncate apex in ventral view, on both lateral sides with numerous fine setae situated linearly on anterior half of distal part of median lobe (Fig. 17B–C). Paramere with very shallow notch on dorsal side, with thin, sparse, inconspicuous proximal setae and thicker, denser, and longer subdistal setae (Fig. 17D).

*Holotype*: TL-H 4.15 mm, TL 4.55 mm, MW 2.25 mm.

*Female*: Without evident differences in external morphology from males, except for not modified pro- and mesotarsi and abdominal ventrite 6 without striae.

#### Distribution.

Papua New Guinea: Central and National Capital District Provinces (Fig. 40).

#### Etymology.

The species is named after Wareaga village. The name is a noun in the nominative singular standing in apposition.

### 15. Exocelina woitapensis

Taxon classificationAnimaliaColeopteraDytiscidae

Shaverdo & Balke sp. n.

http://zoobank.org/B59DD2AA-1304-42DD-86A2-00DA8342BFCC

Figs 6 , 28

Exocelina undescribed sp. MB3399: Toussaint et al. 2014: supplementary figs 1–4, tab. 2.

#### Type locality.

Papua New Guinea: Central Province, Woitape, 08°31.29'S; 147°13.68'E, 1700 m a.s.l.

#### Type material.

*Holotype*: male “Papua New Guinea: Central, Woitape, 1700m, i.2008, 08 31.290S 147 13.684'E, Posman (PNG 166)” (ZSM). *Paratypes*: 2 males, 1 female with the same label as the holotype, the male additionally with a green label “DNA M.Balke 3399” (ZSM). 1 male, 3 females “Papua New Guinea: Central, Woitape, 1500m, i.2008, 08 33.178S 147 15.481E, Posman (PNG 167)”, one female additionally with a green label “DNA M.Balke 3402” (NHMW, ZSM). 1 male “Papua New Guinea: Central, Woitape, 1600m, i.2008, 08 31.581S 147 14.099E, Posman (PNG 165)” (ZSM). 1 female “Papua New Guinea: Central, Kokoda Trek, 590m, i.2008, 09 14.339S 147 36.920E, Posman (PNG 170)” (ZSM).

#### Diagnosis.

Beetle medium-sized; piceous, with reddish brown head and pronotum, later often with darker disc; matt, with strong punctation and microreticulation; male antennae simple; male protarsomere 4 with large, thick, strongly curved anterolateral hook-like seta; median lobe relatively broad, with slightly curved, rounded apex in lateral view and with askew truncate apex in ventral view, on both lateral sides with numerous strong setae situated broad-linearly on whole distal part of median lobe; paramere with small notch on dorsal side. The species is very similar to *Exocelina kabwumensis* sp. n. but differs from it in the larger body size, broader median lobe, and the presence of numerous thick distal setae laterally on the median lobe. It is also similar to *Exocelina andakombensis* sp. n. and *Exocelina injiensis* sp. n. but differs from them in the larger body size, shape of the median lobe, and more numerous and more sparsely situated thick distal setae laterally on the median lobe. From *Exocelina kabwumensis* and *Exocelina andakombensis*, it also differs in the large, thick, strongly curved anterolateral hook-like seta of protarsomere 4. This species co-occurs with *Exocelina posmani* sp. n., see under its diagnosis for their morphological differences.

#### Description.

*Size and shape*: Beetle medium-sized (TL-H 3.6–4.0 mm, TL 4.0–4.35 mm, MW 2.0–2.15 mm), with oblong-oval habitus, broadest at elytral middle. *Coloration*: Head reddish brown to dark brown, with small darker areas posterior to eyes and sometimes brown V-like spot in vertex; pronotum reddish brown, with darker (to piceous) disc; elytra brown to piceous, with narrow reddish sutural lines; head appendages and legs proximally yellowish red, legs distally darker, reddish brown, especially metathoracic legs (Fig. 28). Teneral specimens paler.

*Surface sculpture*: As in *Exocelina andakombensis* sp. n.

*Structures*: Pronotum with distinct lateral bead. Base of prosternum and neck of prosternal process with distinct ridge, slightly rounded anteriorly. Blade of prosternal process lanceolate, relatively broad, slightly convex, and smooth, with distinct lateral bead and few lateral setae; neck and blade of prosternal process evenly jointed. Abdominal ventrite 6 slightly truncate.

*Male*: Antennae simple. Protarsomere 4 with large, thick, strongly curved anterolateral hook-like seta. Protarsomere 5 ventrally with anterior band of ca. 60 and posterior row of 16 relatively long, thin setae (Fig. 6A). Median lobe relatively broad, with slightly curved, elongate, rounded apex in lateral view and with askew truncate apex in ventral view, on both lateral sides with numerous strong setae situated broad-linearly on whole distal part of median lobe (Fig. 6B–C). Paramere with small notch on dorsal side and dense setae on subdistal part; proximal setae inconspicuous (Fig. 6D). Abdominal ventrite 6 with 5–8 lateral striae on each side.

*Holotype*: TL-H 4 mm, TL 4.5 mm, MW 2.15 mm.

*Female*: Without evident differences in external morphology from males, except for not modified pro- and mesotarsi and abdominal ventrite 6 without striae.

#### Distribution.

Papua New Guinea: Central Province (Fig. 40).

#### Etymology.

The species is named after Woitape Village. The name is an adjective in the nominative singular.

### Key to species of the *Exocelina danae*-group

The key is based mostly on male characters. In many cases females cannot be assigned to species due to the similarity of their external and internal structures (for female genitalia see figs 17a and 17b in Shaverdo et al. (2005)). Some species are rather similar on external morphology, therefore, in most cases the male genitalia need to be studied for reliable species identification. Numbers in parentheses refer to the arrangement of the species descriptions above.

**Table:** 

1	Male and female antennomere 2 enlarged, evidently larger than other antennomeres (Figs 24–26)	(***miriae*-subgroup) 2**
–	Male and female antennomeres simple, not modified	**4**
2	Beetle smaller, TL-H: 3.5 mm, reddish-brown, matt dorsally due to strong microreticulation and punctation (Fig. 24)	(2) ***rufa***
–	Beetle larger, TL-H: 3.9–4.5 mm, with darker dorsal coloration, shiny, with evident microreticulation and weak punctation (Figs 25–26)	**3**
3	Median lobe with apex slightly curved, elongate in lateral view and rounded in ventral view; numerous fine setae situated linearly on distal part of median lobe along the lateral sides (Figs 2–3, figs 76, 82 in Balke (1998))	(1) ***miriae***
–	Median lobe with apex slightly curved, broad in lateral view and concave in ventral view, and with a small bunch of fine distal setae on both lateral sides (Fig. 4B–C)	(3) ***tekadu* sp. n.**
4	Beetle matt dorsally due to strong microreticulation and punctation	**5**
–	Beetle shiny, with evident microreticulation and weak punctation	**8**
5	Median lobe narrow, with slightly curved, broad apex and few fine distal setae in lateral view (Fig. 5B–C)	(10) ***kabwumensis* sp. n.**
–	Median lobe broader, with numerous thick setae in lateral view (Figs 6–8)	**6**
6	Beetle larger, TL-H: 3.6–4.0 mm (Fig. 28). Median lobe with more numerous and more sparsely situated thick distal setae laterally; paramere with small dorsal notch (Fig. 6C–D)	(15) ***woitapensis* sp. n.**
–	Beetle smaller, TL-H: 3.05–3.55 mm (Figs 29–30). Median lobe with less numerous and more compactly situated thick distal setae laterally; paramere without notch, slightly concave dorsally (Figs 7C–D, 8C–D)	**7**
7	Protarsomere 4 with weakly curved anterolateral hook-like seta, equal to more laterally situated large seta (Fig. 7A). Median lobe with apex broader in lateral view and slightly concave in ventral view; distal setae of median lobe situated on broader area, especially apically, carina absent (Fig. 7B–C)	(4) ***andakombensis* sp. n.**
–	Protarsomere 4 with large, thick, strongly curved anterolateral hook-like seta (Fig. 8A). Median lobe with apex narrower in lateral view and truncate in ventral view; distal setae of median lobe situated linearly under fine carina (Fig. 8B–C)	(9) ***injiensis* sp. n.**
8	Apex of median lobe more strongly curved in lateral view (Figs 9–18)	**9**
–	Apex of median lobe evenly curved in lateral view (Figs 19–23)	**11**
9	Median lobe distinctly broadened subdistally and narrowed apically, lateral sides like strong, thick folds in ventral view (Figs 9–13)	(6) ***damantiensis***
–	Median lobe with subparallel sides and broader apex, lateral folds inconspicuous (Figs 17B, 18B)	**10**
10	Median lobe robust, with lateral margins apically and subapically thicker, bordered with dorsolateral carina, and with longer apex in lateral view; distal setae of median lobe situated linearly (Fig. 17B–C)	(13) ***wareaga* sp. n.**
–	Median lobe slender, with lateral margins thinner, without dorsolateral carina, and with shorter apex in lateral view; distal setae of median lobe situated on broader, compacter area (Fig. 18B–C)	(14) ***varirata* sp. n.**
11	Beetle smaller, TL-H: 3.4–4.1 mm (Fig. 32). Median lobe smaller, thinner, and narrower (Fig. 19, figs 51, 69 in Balke (1998)); paramere with dorsal notch, its subdistal part larger, with stronger setation (Fig. 19F, fig. 38 in Balke (1998))	(7) ***danae***
–	Beetle larger, TL-H: 3.65–4.75 mm (Figs 35–38). Median lobe larger, thicker, and broader; paramere without notch, slightly concave dorsally, its subdistal part narrower, with weaker setation (Figs 20–23)	**12**
12	Median lobe with broadly pointed apex in ventral view (Fig. 20B). Protarsomere 4 with weakly curved anterolateral hook-like seta, smaller than more laterally situated large seta (Fig. 20A)	(11) ***marawaka* sp. n.**
–	Median lobe with slightly concave apex in ventral view (Figs 21–23). Protarsomere 4 with large or small anterolateral hook-like seta	**13**
13	Median lobe with more elongate, narrower apex in lateral view and lateral sides with numerous fine distal setae almost linearly situated (Fig. 21C)	(8) ***garaina* sp. n.**
–	Median lobe with more rounded, broader apex and lateral sides almost without setae or with fine distal setae situated on broader area, not linearly in lateral view (Figs 22C, 23C)	**14**
14	Median lobe with less rounded apex in lateral view and only some fine distal setae (Fig. 22C). Protarsomere 4 with large, thick, strongly curved anterolateral hook-like seta (Fig. 22A)	(5) ***atrata***
–	Median lobe with distinctly rounded apex in lateral view and with much more numerous fine distal setae (Fig. 23C). Protarsomere 4 with weakly curved anterolateral hook-like seta, equal to more laterally situated large seta (Fig. 23A)	(12) ***posmani* sp. n.**

### Habitats

**Figure 41.**
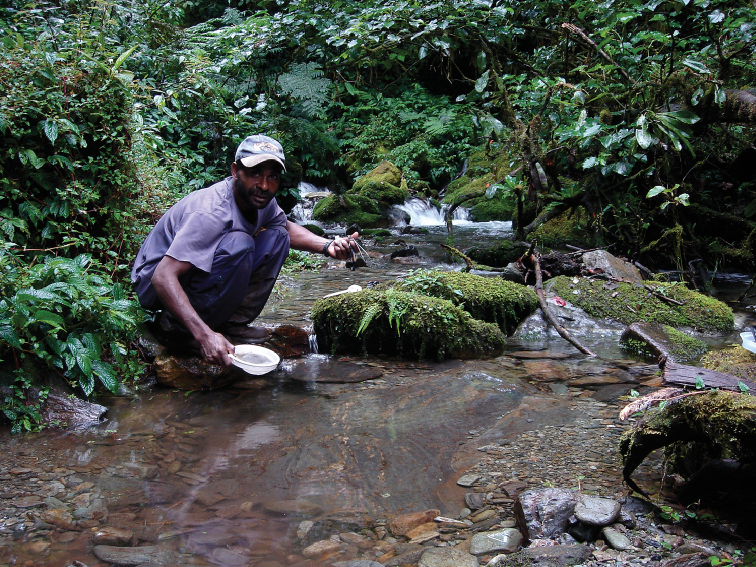
Papua New Guinea, Marawaka area, mid-montane forest stream, with Andrew Kinibel; photo by M. Balke.

All species treated here are associated with running water as almost all previously studied New Guinea *Exocelina* (Shaverdo et al. 2012). Figure 41 shows one of the habitats in the Marawaka area: a mid-montane forest stream with small bays and puddles at its edge, which yielded a large number of these beetles. The following nine species are known from this area: *Exocelina marawaka* sp. n., *Exocelina andakombensis* sp. n., *Exocelina injiensis* sp. n., *Exocelina miriae* (Balke, 1998), *Exocelina hintelmannae* (Shaverdo, Sagata & Balke, 2005), *Exocelina bismarckensis* Shaverdo & Balke, 2014, *Exocelina kisli* Shaverdo & Balke, 2014, *Exocelina craterensis* Shaverdo & Balke, 2014, and *Exocelina kinibeli* Shaverdo & Balke, 2014. The most abundant of them are *Exocelina miriae* and *Exocelina hintelmannae* followed by *Exocelina marawaka* sp. n., *Exocelina andakombensis* sp. n., and *Exocelina injiensis* sp. n.

## Supplementary Material

XML Treatment for Exocelina miriae

XML Treatment for Exocelina rufa

XML Treatment for Exocelina tekadu

XML Treatment for Exocelina andakombensis

XML Treatment for Exocelina atrata

XML Treatment for Exocelina damantiensis

XML Treatment for Exocelina danae

XML Treatment for Exocelina garana

XML Treatment for Exocelina injiensis

XML Treatment for Exocelina kabwumensis

XML Treatment for Exocelina marawaka

XML Treatment for Exocelina posmani

XML Treatment for Exocelina varirata

XML Treatment for Exocelina wareaga

XML Treatment for Exocelina woitapensis

## References

[B1] Balfour-Browne J (1939) On *Copelatus* Er. and *Leiopterus* Steph. (Coleoptera: Dytiscidae) with descriptions of new species. Transactions of the Royal entomological Society of London 88(2): 57–88. doi: 10.1111/j.1365-2311.1939.tb00250.x

[B2] Balke M (1998) Revision of New Guinea *Copelatus* Erichson, 1832 (Insecta: Coleoptera: Dytiscidae): The running water species, Part I. Annalen des Naturhistorischen Museum Wien 100B: 301–341.

[B3] Balke M (1999) Two new species of the genus Copelatus Erichson, 1832, subgenus Papuadytes Balke, 1998, from Papua New Guinea (Insecta: Coleoptera: Dytiscidae). Annalen des Naturhistorischen Museum Wien 101B: 273–276.

[B4] Balke M (2001) Replacement names for three New Guinea species of *Copelatus*, subgenus Papuadytes Balke, 1998 (Coleoptera: Dytiscidae). Annalen des Naturhistorischen Museum Wien 103B: 361–362.

[B5] Balke M Pons J Ribera I Sagata K Vogler AP (2007) Infrequent and unidirectional colonization of hyperdiverse *Papuadytes* diving beetles in New Caledonia and New Guinea. Molecular Phylogenetics and Evolution 42: 505–516. doi: 10.1016/j.ympev.2006.07.019 16979911 10.1016/j.ympev.2006.07.019

[B6] Broun T (1886) Manual of the New Zealand Coleoptera. Parts III and IV. Government Printer, Wellington, 745–973.

[B7] Guéorguiev VB (1968) Essai de classification des coléoptères Dytiscidae. I. Tribus Copelatini (Colymbetinae). Izvestiya na Zoologicheskiya Institut (s Muzei) Sofiya 28: 5–45.

[B8] Guéorguiev VB Rocchi S (1993) Contibuto alla conoscenza dei Dytiscidae della Nuovo Guinea (Coleoptera). Frustula entomologica (1992), n.s. XV(XXVIII): 147–166.

[B9] Guignot F (1956) Dytiscides récoltés par le Dr. L. Biro en nouvelle Guinée at dans l’ile de Java (Coleoptera). Annales historico-naturales Musei nationalis hungarici (N.S.) VII: 51–60.

[B10] Nilsson AN (2001) Dytiscidae. World catalogue of insects. Vol. 3 Apollo Books, Stenstrup, 1–395.

[B11] Nilsson AN (2007) Exocelina Broun, 1886, is the valid name of Papuadytes Balke, 1998. Latissimus 23: 33–34.

[B12] Nilsson AN Fery H (2006) World Catalogue of Dytiscidae – corrections and additions, 3 (Coleoptera: Dytiscidae). Koleopterologische Rundschau 76: 55–74.

[B13] Shaverdo HV Balke M (2014) *Exocelina kinibeli* sp.n. from Papua New Guinea, a new species of the *E. ullrichi*-group (Coleoptera: Dytiscidae). Koleopterologische Rundschau 84: 31–40.

[B14] Shaverdo HV Sagata K Balke M (2005) Five new species of the genus *Papuadytes* Balke, 1998 from New Guinea (Coleoptera: Dytiscidae). Aquatic Insects 27(4): 269–280. doi: 10.1080/01650420500290169

[B15] Shaverdo HV Surbakti S Hendrich L Balke M (2012) Introduction of the *Exocelina ekari*-group with descriptions of 22 new species from New Guinea (Coleoptera, Dytiscidae, Copelatinae). ZooKeys 250: 1–76. doi: 10.3897/zookeys.250.3715 10.3897/zookeys.250.3715 PMC3558971 23378803

[B16] Shaverdo HV Hendrich L Balke M (2013) *Exocelina baliem* sp. n., the only known pond species of New Guinea *Exocelina* Broun, 1886 (Coleoptera, Dytiscidae, Copelatinae). ZooKeys 304: 83–99. doi: 10.3897/zookeys.304.4852 10.3897/zookeys.304.4852 PMC3689123 23794909

[B17] Shaverdo H Sagata K Panjaitan R Menufandu H Balke M (2014) Description of 23 new species of the *Exocelina ekari*-group from New Guinea, with a key to all representatives of the group (Coleoptera, Dytiscidae, Copelatinae). ZooKeys 468: 1–83. doi: 10.3897/zookeys.468.8506 10.3897/zookeys.468.8506 PMC4296520 25610341

[B18] Shaverdo H Panjaitan R Balke M (2016a) A new, widely distributed species of the *Exocelina ekari*-group from West Papua (Coleoptera, Dytiscidae, Copelatinae). ZooKeys 554: 69–85. doi: 10.3897/zookeys.554.6065 10.3897/zookeys.554.6065 PMC4740830 26877680

[B19] Shaverdo H Sagata K Balke M (2016b) Description of two new species of the *Exocelina broschii*-group from Papua New Guinea, with revision and key to all representatives of this species group (Coleoptera, Dytiscidae, Copelatinae). ZooKeys 577: 125–148. doi: 10.3897/zookeys.577.7254 10.3897/zookeys.577.7254 PMC4829886 27110191

[B20] Shaverdo H Panjaitan R Balke M (2016c) *Exocelina ransikiensis* sp. nov. from the Bird’s Head of New Guinea (Coleoptera: Dytiscidae: Copelatinae). Acta Entomologica Musei Nationalis Pragae 56(1): 103–108.

[B21] Toussaint EFA Hall R Monaghan MT Sagata K Ibalim S Shaverdo HV Vogler AP Pons J Balke M (2014) The towering orogeny of New Guinea as a trigger for arthropod megadiversity. Nature Communications 1: 1–10 + 10 supplements, 5: 4001. doi: 10.1038/ncomms5001 10.1038/ncomms5001 24874774

